# (±)-Peniorthoesters A and B, Two Pairs of Novel Spiro-Orthoester en-antiomers With an Unusual 1,4,6-Trioxaspi-ro[4.5]decane-7-One Unit From *Penicillium minioluteum*

**DOI:** 10.3389/fchem.2018.00605

**Published:** 2018-12-07

**Authors:** Xiaorui Liu, Chunmei Chen, Yinyu Zheng, Mi Zhang, Qingyi Tong, Junjun Liu, Qun Zhou, Jianping Wang, Zengwei Luo, Hucheng Zhu, Yonghui Zhang

**Affiliations:** Hubei Key Laboratory of Natural Medicinal Chemistry and Resource Evaluation, School of Pharmacy, Tongji Medical College, Huazhong University of Science and Technology, Wuhan, China

**Keywords:** peniorthoesters, *Penicillium minioluteum*, spiro-orthoester, enantiomers, NO production inhibition activity

## Abstract

(±)-Peniorthoesters A and B (±**1** and ±**2**), two pairs of unprecedented spiro-orthoester enantiomers with a 1,4,6-trioxaspiro[4. 5]decane-7-one unit, were obtained from *Penicillium minioluteum*. Their structures were determined by spectroscopic methods, X-ray diffraction analyses, and ECD calculations. (±)-Peniorthoesters A and B are the first examples of spiro-orthoester enantiomers, and they represent the first spiro-orthoesters originating from fungi. All compounds showed potential inhibitory activities comparable to dexamethasone against NO production with IC_50_ values ranging from 14.2 to 34.5 μM.

## Introduction

Orthoesters, a special functional group characterized by three alkoxy groups attached to a single carbon atom, are unusual structural subunits in natural products (Liao et al., 2009). Natural occurring orthoesters include several major types, such as daphnane diterpenoid orthoesters (He et al., 2002), limonoid orthoesters (Roy and Saraf, 2006), steroid orthoesters (Steyn and van Heerden, 1998), and coumarinoid orthoesters (Santana et al., 2004). In our previous study on the plant Wikstroemia chamaedaphne, three new daphnane type diterpenoids with orthoester group were isolated (Guo et al., 2015). A literature investigation revealed that most of these orthoesters originate from plants, and only a few originate from fungi, such as novofumigatonin, a meroterpenoid orthoester from *Aspergillus novofumigatus* (Rank et al., 2008). As a special class of natural products, orthoesters have attracted great attention due to their diverse structures and biological properties (Liao et al., 2009; Bourjot et al., 2014; Li et al., 2015; Liu et al., 2017).

**Figure 1:**
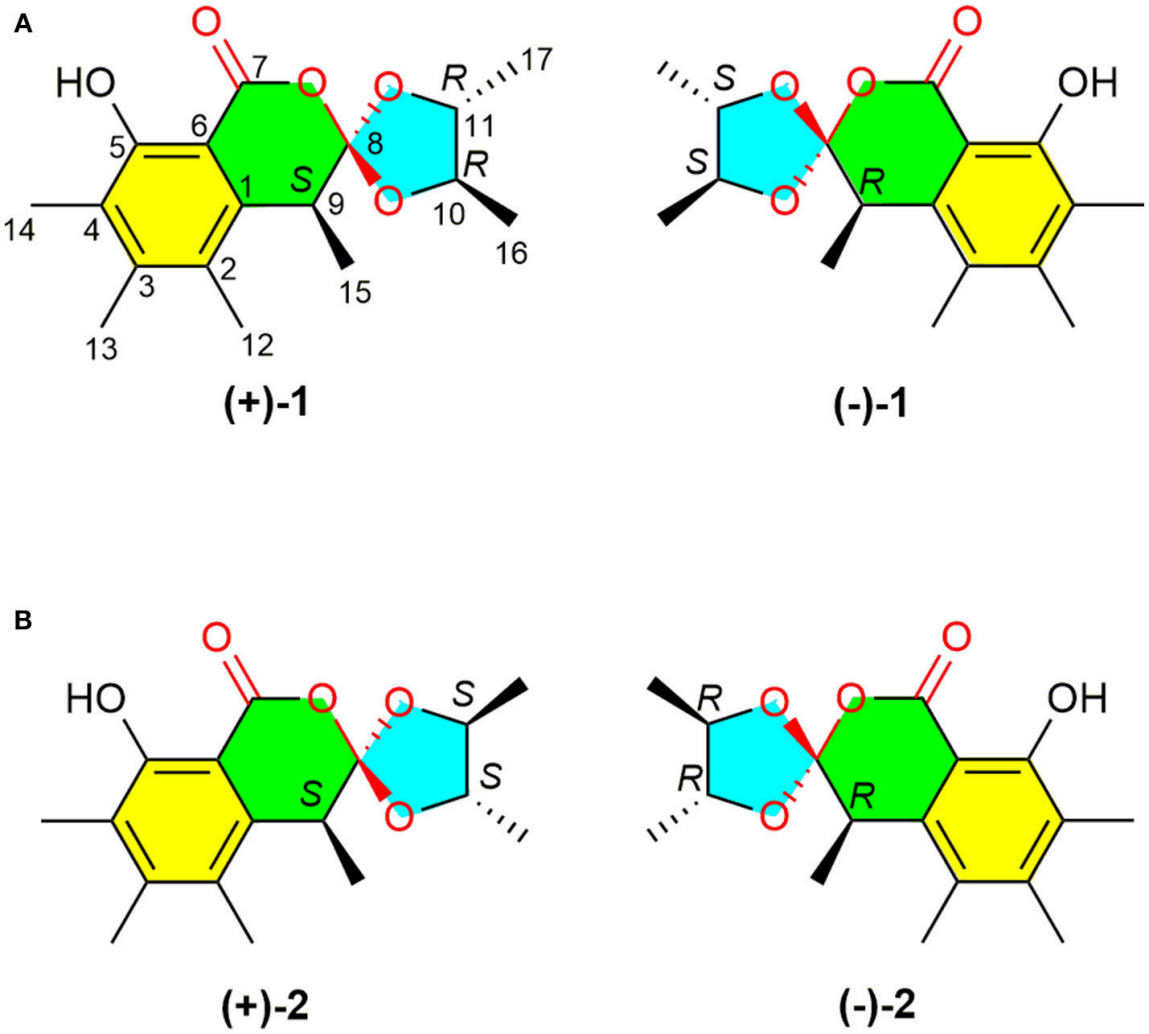
Structures of (±)-peniorthoesters A (±**1**) and B (±**2**).

Fungi have historically played an important role in drug discovery. The genus *Penicillium* has been shown to be a rich source of structurally unique and biologically active secondary metabolites (Meng et al., 2016; Sun et al., 2016; Luo et al., 2017) and many metabolites from *Penicillium* are clinically used drugs with penicillin as a representative compound. Previous studies on the secondary metabolites of *Penicillium minioluteum* have resulted in the identification of scores of bioactive metabolites, including isomeric furanones with cytotoxic activities against HeLa cell lines (Tang et al., 2015), sesquiterpene-conjugated amino acids with cytotoxic activities against HepG2 cells (Ngokpol et al., 2015), and hybrid polyketide-terpenoids (Iida et al., 2008). This fungus was also used to produce clovane derivatives, which are the raw materials for the synthesis of rumphellclovane A (Gontijo de Souza et al., 2012), and an enzyme from this fungus was used in the bioconversion (Kmiecik and Zymanczyk-Duda, 2017). During our ongoing search for structurally unique and biologically interesting constituents from fungi (Zhu et al., 2015; Chen et al., 2017; Zhou et al., 2017), *P. minioluteum*, obtained from China General Micro-biological Culture Collection Center (CGMCC), was phytochemically investigated, and two pairs of new orthoesters (Figure 1, compounds ±**1** and ±**2**) were isolated along with their biosynthetic intermediate, sclerotinin A (**3**) (Xiao et al., 2009). The structures and absolute configurations of (±)-**1** and (±)-**2** were determined by a combination of spectroscopic methods, X-ray diffraction analyses, and ECD calculations. (±)-Peniorthoesters A (±**1**) and B (±**2**) are the first examples of spiro-orthoester enantiomers and represent the first spiro-orthoesters originating from fungi.

## Materials and Methods

### General Experimental Procedures

Optical rotations were determined in acetonitrile and dichloromethane on a PerkinElmer 341 polarimeter. UV spectra were obtained on Varian Cary 50 spectrometer. ECD spectra were obtained with a JASCO J-810 spectrometer. IR spectra were acquired on a Bruker Vertex 70 instrument. NMR spectra were recorded on Bruker AM-400 spectrometers, and the ^1^H and ^13^C NMR chemical shifts were referenced to the solvent or solvent impurity peaks for CD_3_Cl at δ_H_ 7.26 and δ_C_ 77.20. HRESIMS data were acquired in the positive-ion mode on a Thermo Fisher LC-LTQ-Orbitrap XL spectrometer. The crystallographic experiments were performed on a Bruker APEX DUO diffractometer equipped with graphite-monochromated Cu Kα radiation (λ = 1.541 78 Å). Semi-preparative HPLC was carried out on an instrument consisting of an Agilent 1,220 controller, an Agilent 1,220 pump, an Agilent UV detector, with a reversed-phased C18 column (5 μm, 10 × 250 mm, Welch Ultimate XB-C18), an Ultimate SiO_2_ column (5 μm, 10 × 250 mm, Welch Materials, Inc.), and a Chiralpak IC column (5 μm, 4.6 × 250 mm, Daicel Chiral Technologies Co., Ltd., China). Chromatography coloums (CC) were performed on silica gel (200–300 mesh; Qingdao Marine Chemical, Inc., Qingdao, China), Sephadex LH-20 (40–70 μm, Amersham Pharmacia Biotech AB, Uppsala, Sweden), and ODS (50 μm, Merck, Germany). Thin-layer chromatographies (TLC) was performed with RP-C_18_ F_254_ plates (Merck, Germany) and silica gel 60 F_254_ (Yantai Chemical Industry Research Institute).

### Fungal Material and Fermentation

The strain in this work was bought from China General Micro-biological Culture Collection Center (CGMCC). A voucher Specimen was preserved in the herbarium of Huazhong University of Science and Technology, China. The fungal strain was cultured on potato dextrose agar (PDA) at 28°C for 8 days to prepare the seed culture. Then the strain was inoculated into 200 Erlenmeyer flasks (1 L each) which had previously been sterilized by autoclaving. Each flask contained 250 g rice and 200 mL distilled water. The flasks were incubated at 20°C for 26 days.

### Extraction and Isolation

The fermented rice substrate was extracted six times in 95% aqueous EtOH at room temperature. The 95% aqueous EtOH extracts were concentrated under vacuum to afford a residue (1.5 kg). The residue was suspended in H_2_O and successively partitioned with petroleum ether and EtOAc. The EtOAc partition fraction (630.0 g) was subjected to a silica gel chromatograph column (CC) using petroleum ether–EtOAc and EtOAc–MeOH gradient elution to give five fractions. Fraction 2 (20.0 g) was chromatographed on C18 reversed phase (RP-18) silica gel CC (gradient elution of MeOH–H_2_O, 20:80–100:0) to give seven subfractions, named Fr. 2A−2G. Fr.2F was successively separated via Sephadex LH-20 CC (CH_2_Cl_2_-MeOH, 1:1) and further purified by RP-C_18_ HPLC to afford compounds **1** and **2** (MeCN–H_2_O, 45:55, 3.5 ml/min, **1** at *t*_R_ 53.2 min, 6.0 mg; **2** at *t*_R_ 56.7 min, 5.1 mg). Subsequently, the separation of **1** by chiral HPLC using a Daicel IC column eluting with isopropanol–n-hexane (5:95) afforded (+)-**1** (*t*_R_ 28.0 min, 2.1 mg) and (–)-**1** (*t*_R_ 30.1 min, 2.0 mg). The enantiomers (+)-**2** (*t*_R_ 8.2 min, 1.6 mg) and (–)-**2** (*t*_R_ 11.1 min, 2.7 mg) were also obtained by chiral HPLC using a Daicel IC column eluting with isopropanol–*n*-hexane (10:90).

*Compounds (*±*)-****1***: white powder; [α] ±0 (c 0.4, MeCN); UV (MeCN) λ_max_ (log ε) 216 (4.50), 259 (4.23), 329 (4.04) nm; IR (KBr) ν_max_ 3,435, 2,982, 2,936, 1,661, 1,612, 1,456, 1,420, 1,383, 1,341, 921, 883, 809, 753 cm^−1^; ^1^H NMR (CDCl_3_, 400 MHz) and ^13^C NMR (CDCl_3_, 100 MHz) data, see Table 1; HRESIMS *m*/*z* 307.1538 [M + H]^+^ (calcd for C_17_H_23_O_5_, 307.1545).
*(*+*)-****1***: white amorphous powder; [α${]}_{\text{D}}^{25}$ +37 (c 0.1, CH_2_Cl_2_); ECD (MeCN) 229 (Δε +1.63), 254 (Δε +4.75), 325 (Δε −0.99) nm.
*(*–*)-****1***: white amorphous powder; [α${]}_{\text{D}}^{25}$ −36 (c 0.1, CH_2_Cl_2_); ECD (MeCN) 229 (Δε −1.66), 254 (Δε −4.34), 325 (Δε +1.23) nm.
*Compounds (*±*)-****2***: white powder; [α${]}_{\text{D}}^{25}$ ±0 (c 0.3, MeCN); UV (MeCN) λ_max_ (log ε) 216 (4.60), 259 (3.99), 329 (3.71) nm; IR (KBr) ν_max_ 3,435, 2,980, 2,932, 1,669, 1,612, 1,456, 1,421, 1,379, 1,339, 920, 881, 810, 763 cm^−1^; ^1^H NMR (CDCl_3_, 400 MHz) and ^13^C NMR (CDCl_3_, 100MHz) data, see Table 1; HRESIMS *m*/*z* 307.1534 [M + H]^+^ (calcd for C_17_H_23_O_5_, 307.1545).
*(*+*)-****2***: white amorphous powder; [α${]}_{\text{D}}^{25}$ +30 (c 0.1, CH_2_Cl_2_); ECD (MeCN) 224 (Δε +2.37), 258 (Δε +4.64), 319 (Δε −0.84) nm.
*(*–*)-****2***: white amorphous powder; [α${]}_{\text{D}}^{25}$ −30 (c 0.1, CH_2_Cl_2_); ECD (MeCN) 224 (Δε −2.42), 258 (Δε −6.58), 319 (Δε +1.60) nm.

**Table 1:** ^1^H and ^13^C NMR Spectroscopic Data for **1** and **2** (in CDCl_3_).

**No**.	**1**			**2**		
	**δ_**C**_**	**type**	**δ_**H**_ (mult., *J* in Hz)**	**δ_**C**_**	**type**	**δ_**H**_ (mult., *J* in Hz)**
1	137.8	C			137.6	C
2	123.5	C			123.6	C
3	145.7	C			145.7	C
4	123.7	C			123.8	C
5	158.8	C			158.7	C
6	103.5	C		103.7	C	
7	170.3	C		170.2	C	
8	124.0	C		124.0	C	
9	38.4	CH	3.31 q (7.1)	38.5	CH	3.33 q (7.0)
10	81.7^a^	CH	3.91 dq (8.5, 6.1)^a^	81.1^a^	CH	3.84 dq (8.1, 6.2)^a^
11	79.3^a^	CH	4.18 dq (8.5, 6.1)^a^	79.8^a^	CH	4.36 dq (8.1, 6.2)^a^
12	14.7	CH_3_	2.17 s	14.6	CH_3_	2.17 s
13	17.5	CH_3_	2.25 s	17.4	CH_3_	2.24 s
14	11.9	CH_3_	2.20 s	11.9	CH_3_	2.20 s
15	16.9	CH_3_	1.30 d (7.1)	17.2	CH_3_	1.30 brd (6.3)
16	16.7^a^	CH_3_	1.47 d (6.1)^a^	16.7^a^	CH_3_	1.30 brd (6.1)^a^
17	18.4^a^	CH_3_	1.25 d (6.1)^a^	18.6^a^	CH_3_	1.39 d (6.1)^a^
HO-5			11.41 s			11.41 s

a *Interchangeable assignments between the two CHCH_3_ groups*.

### Computational Details

The crystal structure of 9*R*,10*S*,11*S***-1**, and 9*R*,10*R*,11*R-***2** were optimized at the B3LYP/6-31G(d) level in acetonitrile with the IEFPCM solvation model using Gaussian 09 program. The harmonic vibrational frequencies were calculated to confirm the stability of the optimized structure. The electronic circular dichroism (ECD) spectrum were calculated using the TDDFT methodology at the LC-wPBE/6-311++G(d,p) level with acetonitrile as solvent by the IEFPCM solvation model implemented in Gaussian 09 program. The ECD spectra was simulated using a Gaussian function with a bandwidth σ of 0.3 eV. The UV correction was applied to generate the final spectra (Zhu, 2015).

### Single-Crystal X-ray Diffraction Analysis and Crystallographic Data

Crystallographic data of compound **1** (CCDC 1840165): C_17_H_22_O_5_, *M* = 306.34, monoclinic, *T* = 297(2) K, λ = 1.54178 Å, colorless platelet (crystallized from distilled water at room temperature), size 0.12 × 0.10 × 0.10 mm3, *a* = 11.7937(4) Å, *b* = 32.5593(12) Å, *c* = 8.1659(3) Å, α = 90.00°, β = 91.95(2)°, γ = 90.00°, *V* = 3,133.84(19) Å3, space group *P*21/c, *Z* = 8, *Dc* = 1.299 g/cm^3^, μ(CuKα) = 0.782 mm^−1^, *F*_(000)_ = 1312, 48082 reflections and 5,729 independent reflections (*R*_int_ = 0.0528) were collected in the θ range of 2.71° ≤ θ ≤ 69.99° with index ranges of *h*(−14/14), *k*(−39/39), *l*(−9/9), completeness θmax = 98%, data/restraints/parameters 5,729/0/412. Largest difference peak and hole = 0.257 and −0.184 e Å−3. The final *R*_1_ values were 0.0489 (*I* > 2σ(*I*)). The final *wR*(*F*^2^) values were 0.1364 (*I* > 2σ(*I*)). The final *R*_1_ values were 0.0521 (all data). The final *wR*(*F*^2^) values were 0.1381 (all data). The goodness of fit on *F*^2^ was 1.045.

Crystallographic data of compound **2** (CCDC 1840166): C_17_H_22_O_5_, *M* = 306.34, monoclinic, *T* = 297(2) K, λ = 1.54178 Å, colorless platelet (crystallized from distilled water at room temperature), size 0.12 × 0.10 × 0.10 mm3, *a* = 7.4709(2) Å, *b* = 8.9377(12) Å, *c* = 13.5720(3) Å, α = 92.43°, β = 100.25(2)°, γ = 113.76°, *V* = 809.60(19) Å3, space group *P*-1, *Z* = 2, *Dc* = 1.257 g/cm^3^, μ(CuKα) = 0.757 mm^−1^, *F*_(000)_ = 328, 14,855 reflections and 2,814 independent reflections (*R*_int_ = 0.0374) were collected in the θ range of 5.45° ≤ θ ≤ 70.86° with index ranges of *h*(−8/7), *k*(−10/10), *l*(−16/16), completeness θmax = 95%, data/restraints/parameters 2,814/0/207. Largest difference peak and hole = 0.232 and −0.241 e Å−3. The final *R*_1_ values were 0.0588 (*I* > 2σ(*I*)). The final *wR*(*F*^2^) values were 0.1750 (*I* > 2σ(*I*)). The final *R*_1_ values were 0.0647 (all data). The final *wR*(*F*^2^) values were 0.1855 (all data). The goodness of fit on *F*^2^ was 1.076.

### Determination of No Production

RAW 264.7 cells were obtained from the Boster Biological Technology Co., Ltd (Wuhan, China) and maintained in DMEM containing 10% heat-inactived fetal bovine serum (FBS) (Gibco BRL Co, Grand Island, NY, United States) at 37°C in humidified incubator containing 5% CO_2_. All tested compounds were dissolved in DMSO (the final concentration of DMSO was <0.25% in assay). RAW 264.7 cells were seeded into 48-well plates (1 × 10^5^cells/well) for 24 h and then pretreated with different concentrations (1–40 μM) of test compounds. After being incubated for 3 h, the cells were stimulated with 100 ng/ml LPS (final concentration) for another 24 h. Dexamethasone was used as the positive control in the experiment. NO content in the supernatant was measured using Griess reagent. The absorbance at 540 nm was measured on a microplate reader. All the experiments were performed in three independent replicates.

### Cytotoxic Activity

Cell lines were cultured in RPMI-1640 or DMEM medium (HyClone, USA), supplemented with 10% fetal bovine serum (HyClone, USA) at 37°C in a humidified atmosphere with 5% CO_2_. For cell viability assay, cells were plated into 96-well plates in 50 μl of medium and then compounds were serially diluted in medium and delivered to the cells as 2 × solutions in 50 μl of medium. After 48 h, cell viability was detected by a CCK-8 Kit (Dojindo,Japan) according to the instruction. Growth relative to untreated cells was calculated (positive control, anticancer drug VP16), and this data was used for the dose-response curve, the IC_50_ (50% inhibiting concentration) of compounds to each cell lines were calculated by SPSS.

## Results and Discussions

Compound **1** was isolated as a white powder. Its UV spectrum exhibited absorption maxima at 216 and 260 nm. Its IR spectrum indicated the presence of an OH functionality (3,435 cm^−1^), a conjugated carbonyl group (1,661 cm^−1^), and an aromatic ring (1,612 and 1,456 cm^−1^). The molecular formula of **1** was determined to be C_17_H_22_O_5_ by HRESIMS with an [M + H]^+^ ion peak at *m*/*z* 307.1538 (calcd for C_17_H_23_O_5_, 307.1545), implying seven degrees of unsaturation. The ^1^H NMR spectroscopic data of **1** (Table 1) revealed the presence of two oxygenated methines [δ_*H*_ 4.18 (1H, dq, *J* = 8.5, 6.1 Hz, H-11) and 3.91 (1H, dq, *J* = 8.5, 6.1 Hz, H-10)], one sp^3^ methine [δ_H_ 3.31, 1H, q, *J* = 7.1 Hz, H-9], and six methyl groups [δ_H_ 1.47 (d, *J* = 6.1 Hz, H_3_-16), 1.30 (d, *J* = 7.1 Hz, H_3_-15), 1.25 (d, *J* = 6.1 Hz, H_3_-17), 2.17 (s, H_3_-12), 2.20 (s, H_3_-14), and 2.25 (s, H_3_-13)]. The ^13^C NMR spectrum of **1** exhibited signals assignable to a conjugated carbonyl (δ_C_ 170.3), a hexa-substituted benzene ring [δ_C_ 158.8, 145.7, 137.8, 123.7, 123.5, and 103.5], one oxygenated quaternary carbon (δ_C_ 124.0), six methyl groups and three methines (including two oxygenated ones). The above analyses confirmed the presence of an ester carbonyl group and a hexa-substituted benzene ring, which account for five degrees of unsaturation, indicating the presence of two additional rings. With the aid of the HSQC spectrum, all protons were unambiguously assigned to their respective carbons.

**Figure 2:**
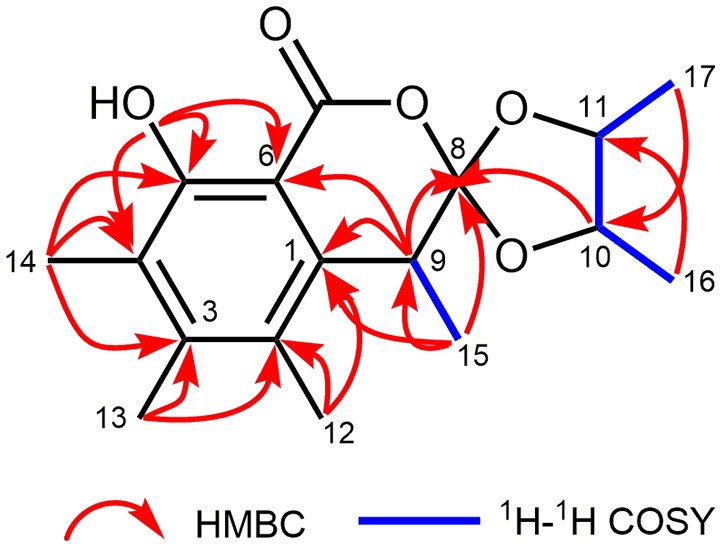
Key 1H−1H COSY and HMBC correlations of **1**.

The planar structure of **1** was elucidated on the basis of ^1^H–^1^H COSY and HMBC experiments (Figure 2). The HMBC spectrum of **1** displayed correlations from H_3_-14 to C-3, C-4, and C-5; from H_3_-13 to C-2, C-3, and C-4; from H_3_-12 to C-1, C-2, and C-3; and from H-9 to C-1, and C-6, which together with the HMBC correlations from the OH to C-4, C-5, and C-6 constructed the hexa-substituted benzene ring. In addition, two spin systems of H_3_-17/H-11/H-10/H_3_-16 and H-9/H_3_-15 were established from the ^1^H–^1^H COSY spectrum. Therefore, the HMBC correlations from H-9 to C-1, C-6, and C-8 and from H_3_-15 to C-1, C-8, and C-9 suggested the C-15/C-9/C-8 fragment was connected to the benzene ring via C-9. Moreover, the ester carbonyl (δ_C_ 170.3) was connected to C-6 based on the chemical shifts of C-6 (δ_C_ 103.5), C-1 (δ_C_ 137.8), and C-3 (δ_C_ 145.7). Combined with the chemical shifts of C-10 (δ_C_ 81.7) and C-11 (δ_C_ 79.3), the C-17/C-11/C-10/C-16 fragment was proposed to be a 2,3-butanediol unit, which should be linked with C-8 and form a 4,5-dimethyl-1,3-dioxolane moiety. Finally, a lactone ring was proposed between C-7 and C-8 to satisfy the above deduced tricyclic ring system as well as the chemical shift of C-8 (δ_C_ 124.0). This planar structure satisfied all of the correlations observed in the 2D NMR spectra and the chemical shifts in the 1D NMR spectra.

**Figure 3:**
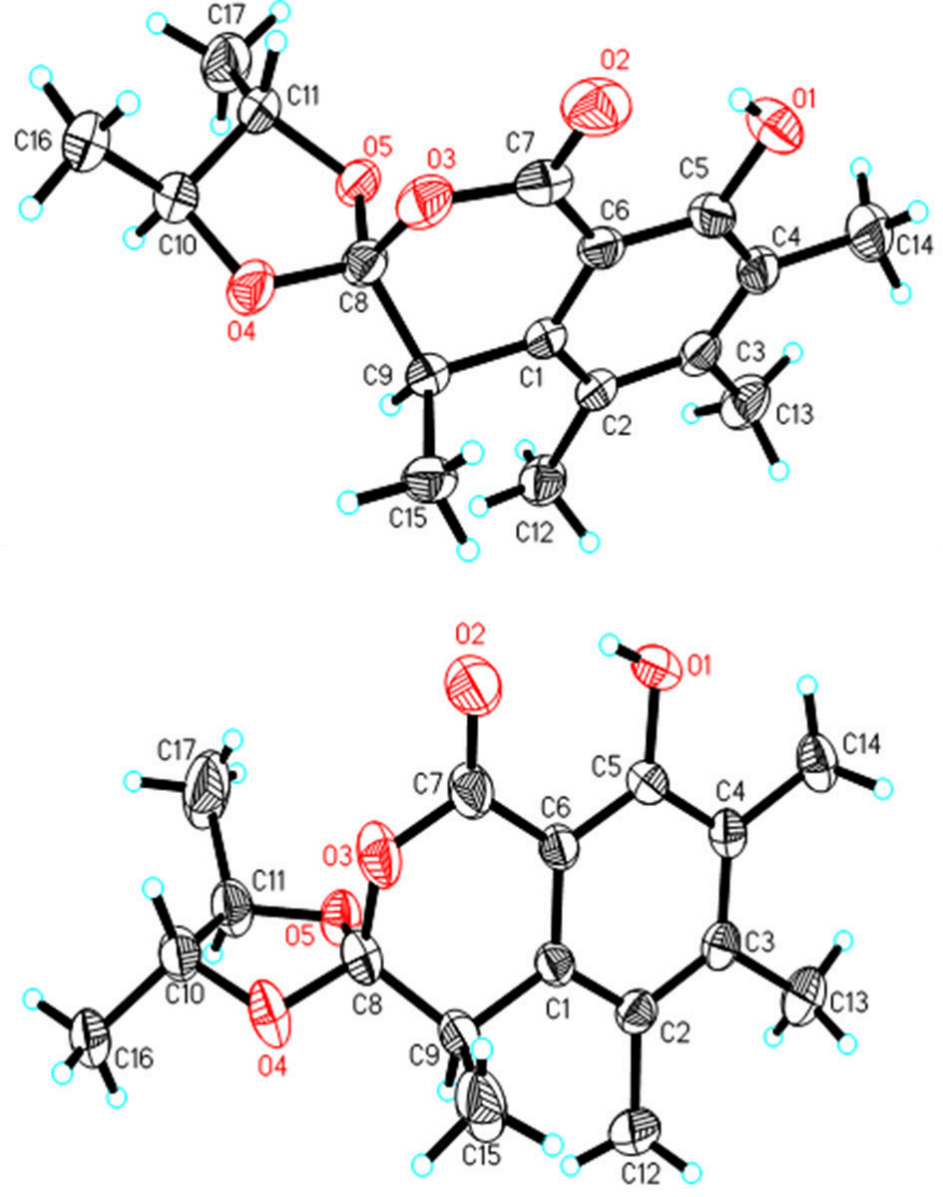
X-ray structures of **1** and **2**.

**Figure 4:**
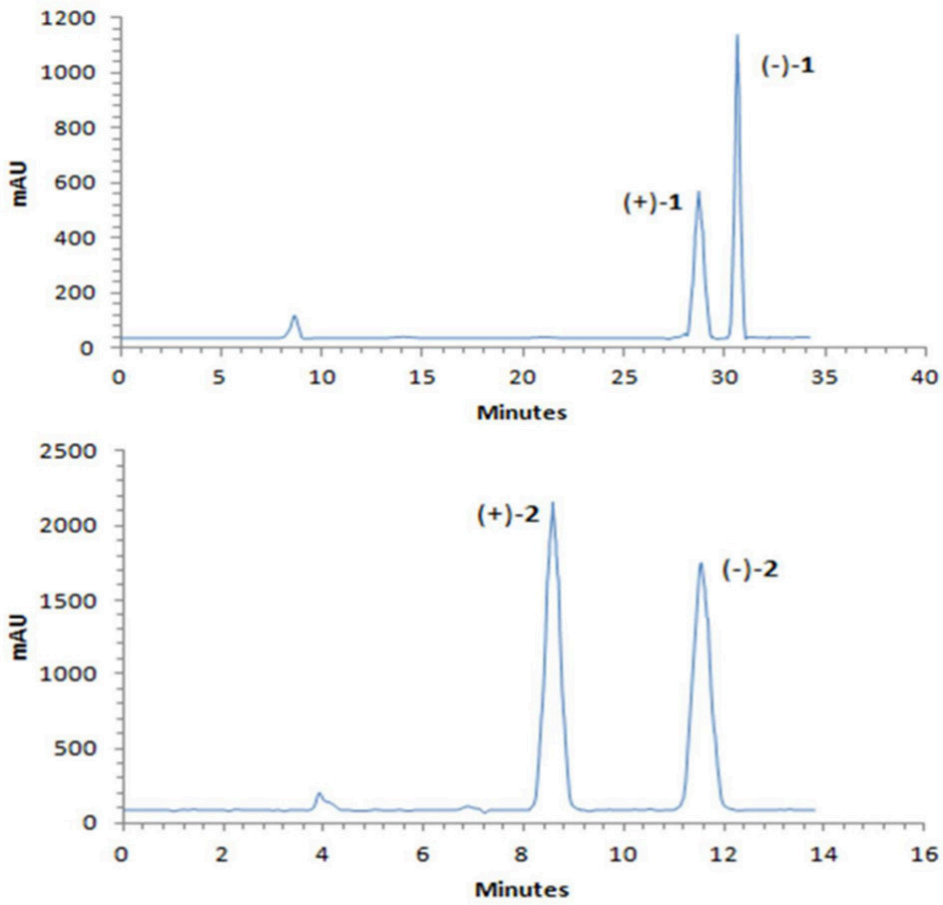
Chiral HPLC separation profiles of (±)-**1** and (±)-**2**.

**Figure 5:**
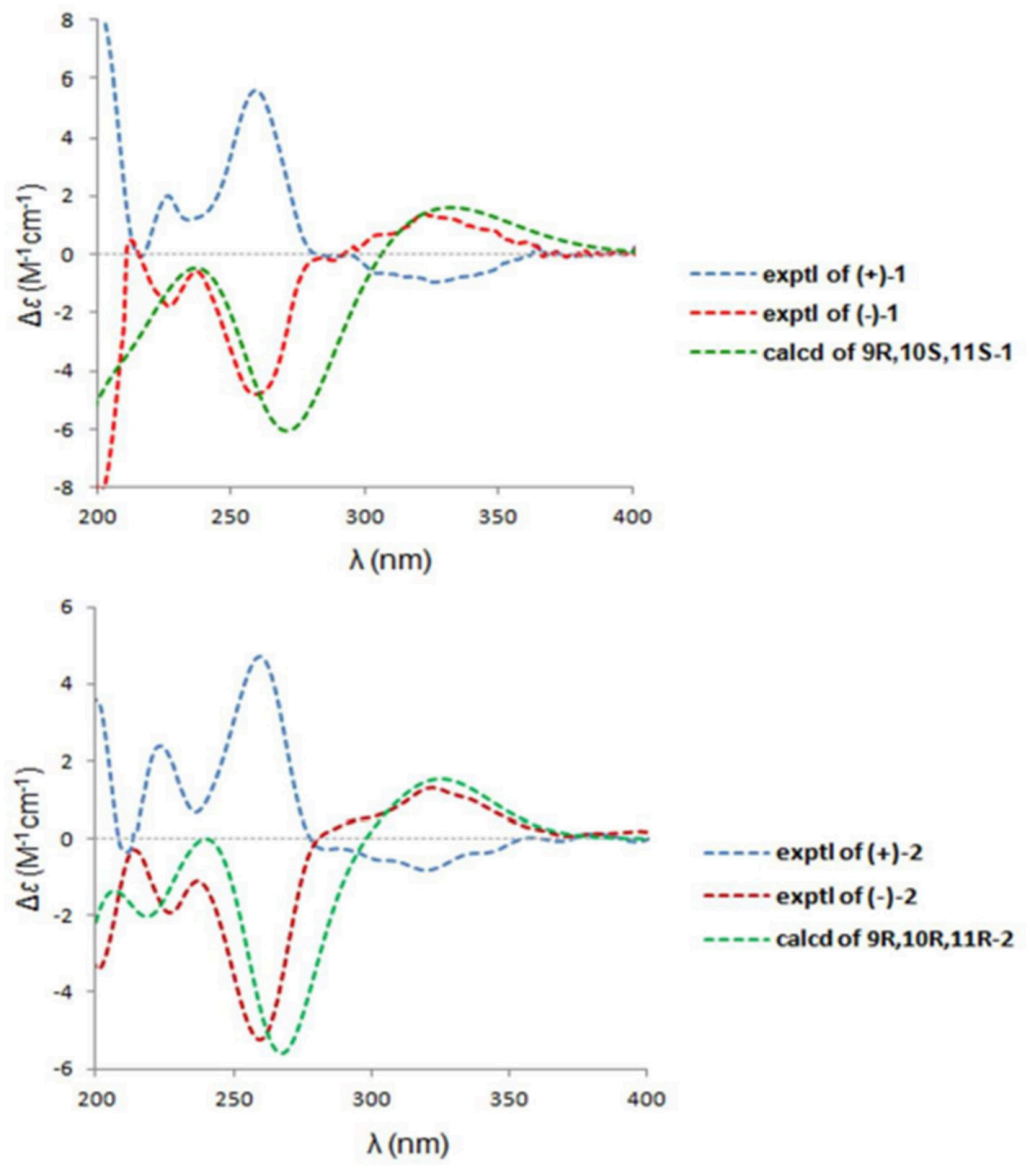
The experimental ECD spectra of (±)-**1** and (±)-**2** and the cal-culated ECD curves.

A NOESY experiment was performed on **1**, but no interactions useful for determining the relative configuration were observed. Unfortunately, the relative configuration of H-10 and H-11 could also not be determined from their coupling constants because they were located on a five-membered ring. To confidently assign the configuration of **1**, we tried to crystallize it so we could use X-ray single-crystal analysis. After a number of attempts, a high-quality single-crystal of **1** was finally obtained from a mixture of methyl alcohol and water. The X-ray crystallography data (CCDC 1840165) obtained with Cu Kα radiation confirmed the structure of **1** (Figure 3). However, because it has a centrosymmetric monoclinic space group of chiral P2_1_/c, indicating the crystal is racemic, the absolute configuration of **1** could not be determined. After analyses by using various types of chiral columns, the racemic nature of this solution was further confirmed by the presence of two peaks in the HPLC chromatogram acquired using a chiral Daicel IC column (Figure 4). Finally, compounds (+)-**1** and (–)-**1** were successfully obtained, and they showed specific rotations with opposite signs {(+)-**1**: [α${]}_{\text{D}}^{20}+$37 (c 0.1, CH_2_Cl_2_); (–)-**1**: [α${]}_{\text{D}}^{20}$-36 (c 0.1, CH_2_Cl_2_)}. In addition, the ECD spectra of (+)-**1** and (–)-**1** displayed similar signal intensities but mirror-image Cotton effects (Figure 5).

The absolute configurations of the two enantiomers of (±)-**1** were further determined by comparing their experimental ECD spectra with those predicted by time-dependent density functional theory (TDDFT) calculations at the B3LYP/6-31G(d) level. As shown in Figure 5, the calculated ECD curve of 9*R*,10*S*,11*S*-**1** displayed good agreement with the experimental curve of (–)-**1**. Therefore, the absolute configurations of (+)-**1** and (–)-**1** were elucidated as 9*S*,10*R*,11*R* and 9*R*,10*S*,11*S*, respectively.

Compound **2**, obtained as a white powder, possesses the same molecular formula (C_17_H_22_O_5_) as that of compound **1** based on its HRESIMS data with an [M + H]^+^ ion peak at *m/z* 307.1534 (calcd for C_17_H_23_O_5_, 307.1545). A detailed comparison of its NMR spectroscopic data with those of **1** indicated that the main differences between **1** and **2** were tiny changes in the chemical shifts of C-10 and C-11 as well as their protons [δ_C_ 81.1 (C-10), 79.8 (C-11); δ_H_ 3.84 (1H, dq, *J* = 8.1, 6.2 Hz, H-10), 4.36 (1H, dq, *J* = 8.1, 6.2 Hz, H-11) in **2**; δ_C_ 81.7 (C-10), 79.3(C-11); δ_H_ 3.91 (1H, dq, *J* = 8.5, 6.1 Hz, H-10), 4.18 (1H, dq, *J* = 8.5, 6.1 Hz, H-11) in **1**]. These findings, combined with the 2D NMR data, implied that **2** has the same planar structure as **1**, and it should be a stereoisomer of **1**. The planar structure of **2** was further confirmed by analyses of its ^1^H–^1^H COSY and HMBC spectra. Unfortunately, the NOESY experiment of **2** also did not show any NOESY correlations useful in for the elucidation of the relative configuration of compound **2**.

Similarly, after many attempts, we finally determined the relative configuration of compound **2** by X-ray crystallography analysis with Cu Kα radiation (Figure 3, CCDC 1840166). This single-crystal is triclinic with space group of chiral P-1, also indicating it is racemic. Compound **2** was then separated into a pair of enantiomers by a method similar to what was used for compound **1** (Figure 4), and the enantiomers showed opposite optical rotations {(+)-**2**: [α${]}_{\text{D}}^{20}+$30 (c 0.1, CH_2_Cl_2_); (–)-**2**: [α${]}_{\text{D}}^{20}$-30 (c 0.1, CH_2_Cl_2_)} and mirror image ECD curves (Figure 5). The absolute configurations of the two enantiomers of **2** were further determined by ECD calculations. As shown in Figure 5, the calculated ECD curve of 9*R*,10*R*,11*R*-**2** closely resembled the experimental curve of (–)-**2**, and the absolute configurations of (+)-**2** and (–)-**2** were elucidated as 9*S*,10*S*,11*S* and 9*R*,10*R*,11*R*, respectively.

**Figure 6:**
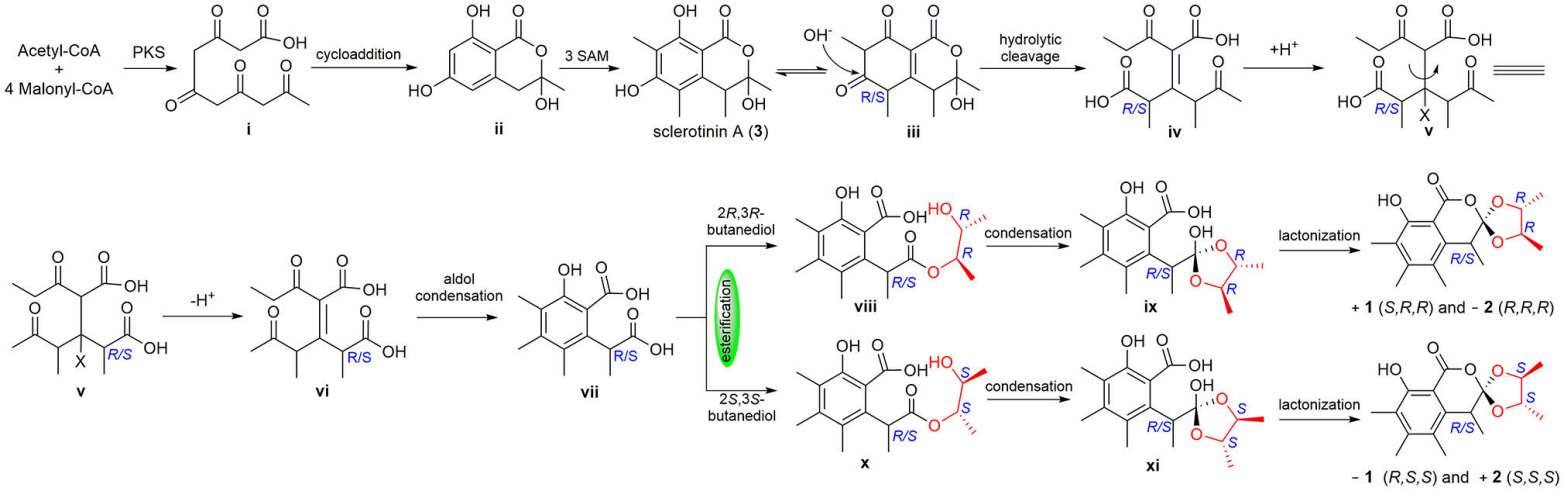
Proposed biosynthetic pathway of **1** and **2**.

To the best of our knowledge, (±)-**1** and (±)-**2** are the first examples of spiro-orthoester enantiomers with an unusual 1,4,6-trioxaspiro[4.5]decane-7-one unit, and they represent the first spiro-orthoesters originating from fungi. The proposed biosynthetic pathway of **1** and **2** was outlined in Figure 6. First, the condensation of acetyl-CoA and four molecules of malonyl-CoA by a polyketide synthase formed **i**, which underwent cycloaddition and methylations to form precursor sclerotinin A (**3**). Then, sclerotinin A underwent isomerization and hydrolytic cleavage to afford **iv**, which further formed **vi** by a H^+^ mediated double bond isomerization. After that, intermediate **vii** was produced by an aldol condensation, which further generated the key intermediates **viii** and **x** via an esterification reaction with 2*R*,3*R*-butanediol and 2*S*,3*S*-butanediol (Ji et al., 2011), respectively. Finally, compounds (±)-**1** and (±)-**2** were formed via condensation and lactonization reactions.

Compounds (±)-**1** and (±)-**2** were tested for their inhibitory activities against NO production in lipopolysaccharide (LPS)-induced RAW264.7 cells. The results revealed that (+)-**1**, (–)-**1**, (+)-**2**, and (–)-**2** exhibited potential inhibitory activities with IC_50_ values of 34.5, 29.6, 23.5, and 15.2 μM, respectively (Table 2). Interestingly, for both pairs of enantiomers, the levorotatory compounds (–**1** and –**2**) showed better inhibitory effects than the dextrorotatory compounds (+**1** and +**2**). Moreover, both (+)-**2** and (–)-**2** showed better inhibitory effects than those of (+)-**1** and (–)-**1** as well as the positive control, dexamethasone. We also tested cytotoxicity of these compounds, but even at the concentration of 40 μM, none of them showed cytotoxicity activity.

**Table 2:** Inhibition of LPS-Induced NO Production.

**Compound**	**IC_**50**_ (μM)**
(**+**)**-**1	34.5
(–)**-**1	29.6
(+)**-**2	23.5
(–)**-**2	14.2
Dexamethasone	27.1

## Conclusion

In conclusion, two pairs of new spiro-orthoester enantiomers, (±)-peniorthoesters A and B (±**1** and ±**2**), were isolated from *P. minioluteum*. These compounds, characterized by an unexpected 1,4,6-trioxaspi-ro[4.5]decane-7-one unit, are the first examples of spiro-orthoester enantiomers, and they represent the first spiro-orthoesters originating from fungi. All of them showed potential inhibitory activities against NO production in activated macrophages with IC_50_ values ranging from 14.2 to 34.5 μM, which are comparable to the positive control, dexamethasone. Their highly functionalized structures and promising biological activities will attract considerable attention from the pharmacological, synthetic, and biosynthetic communities.

## Data Availability Statement

The raw data supporting the conclusions of this manuscript will be made available by the authors, without undue reservation, to any qualified researcher.

## Author Contributions

XL and CC conducted the main experiments and wrote the manuscript. YiZ, MZ, and QZ carried out bioassays. JL did the ECD calculations. JW and ZL analyzed the spectroscopic data. HZ and YoZ initiated and oversaw all research. All authors reviewed the manuscript.

### Conflict of Interest Statement

The authors declare that the research was conducted in the absence of any commercial or financial relationships that could be construed as a potential conflict of interest.

## Supplementary Material

The Supplementary Material includes Full NMR, HRESIMS, UV, and IR spectra of 1 and 2; detailed of the ECD calculations of 1 and 2; X-ray data of 1 and 2 (PDF); and crystallographic data (CIF).
