# Supplementary material for: (±)-Peniorthoesters A and B, Two Pairs of Novel Spiro-Orthoester en-antiomers With an Unusual 1,4,6-Trioxaspi-ro[4.5]decane-7-One Unit From Penicillium minioluteum
Source: Front Chem. 2018 Dec 7;6:605. doi: 10.3389/fchem.2018.00605 (PMC6292945; doi:10.3389/fchem.2018.00605)
Supplement: Supplementary file 1 [file Table_1.DOC]

**(±)-****Peniorthoesters A and B, Two** **Pairs of Novel Sp****iro-****orthoester enantiomers** **with** **an unusual 1,4,6-trioxaspiro[4.5]decane-7-one unit from *Penicillium minioluteum*†**

Xiaorui Liu,‡ Chunmei Chen,‡ Yinyu Zheng, Mi Zhang, Junjun Liu, Qun Zhou, Jianping Wang, Zengwei Luo, Hucheng Zhu,* and Yonghui Zhang*

*Hubei Key Laboratory of Natural Medicinal Chemistry and Resource Evaluation, School of Pharmacy, Tongji Medical College, Huazhong University of Science and Technology, Wuhan 430030, China*

*† Electronic Supplementary Information (ESI) available: Full NMR, HRESIMS, UV, and IR spectra of* ***1*** *and* ***2****; detailed of the ECD calculations of* ***1*** *and* ***2****; X-ray data of* ***1*** *and* ***2*** *(PDF); and crystallographic data (CIF) are included.*

*Corresponding Authors’ Emails:*

*zhangyh@mails.tjmu.edu.cn (Y.Z.); zhuhucheng@hust.edu.cn (H.Z.)*

*‡ These authors contributed equally to this work.*

**Contents**

[**Figure S1.** ^1^H NMR spectrum (400 MHz) of compound **1** in CDCl_3_ 3](#__RefHeading___Toc511173879)

[**Figure S2.** ^13^C NMR spectrum (100 MHz) of compound **1** in CDCl_3_ 3](#__RefHeading___Toc511173880)

[**Figure S3.** HSQC spectrum of compound **1** in CDCl_3_ 4](#__RefHeading___Toc511173881)

[**Figure S4.** HMBC spectrum of compound **1** in CDCl_3_ 4](#__RefHeading___Toc511173882)

[**Figure S5.** ^1^H-^1^H COSY spectrum of compound **1** in CDCl_3_ 5](#__RefHeading___Toc511173883)

[**Figure S6.** NOESY spectrum of compound **1** in CDCl_3_ 5](#__RefHeading___Toc511173884)

[**Figure S7.** HRESIMS spectrum of compound **1** 6](#__RefHeading___Toc511173885)

[**Figure S8.** UV spectrum of compound **1** 6](#__RefHeading___Toc511173886)

[**Figure S9.** IR spectrum of compound **1** 7](#__RefHeading___Toc511173887)

[**Figure S10.** ^1^H NMR spectrum (400 MHz) of compound **2** in CDCl_3_ 7](#__RefHeading___Toc511173888)

[**Figure S11.** ^13^C NMR spectrum (100 MHz) of compound **2** in CDCl_3_ 8](#__RefHeading___Toc511173889)

[**Figure S12.** HMQC spectrum of compound **2** in CDCl_3_ 8](#__RefHeading___Toc511173890)

[**Figure S13.** HMBC spectrum of compound **2** in CDCl_3_ 9](#__RefHeading___Toc511173891)

[**Figure S14.** ^1^H-^1^H COSY spectrum of compound **2** in CDCl_3_ 9](#__RefHeading___Toc511173892)

[**Figure S15.** NOESY spectrum of compound **2** in CDCl_3_ 10](#__RefHeading___Toc511173893)

[**Figure S16.** HRESIMS spectrum of compound **2** 10](#__RefHeading___Toc511173894)

[**Figure S17.** UV spectrum of compound **2** 11](#__RefHeading___Toc511173895)

[**Figure S18.** IR spectrum of compound **2** 11](#__RefHeading___Toc511173896)

[**Figure S19.** Crystal packing of compound **1** 12](#__RefHeading___Toc511173897)

[**Figure S20.** Crystal packing of compound **2** 12](#__RefHeading___Toc511173898)


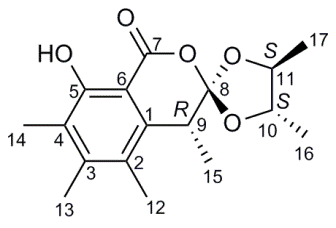

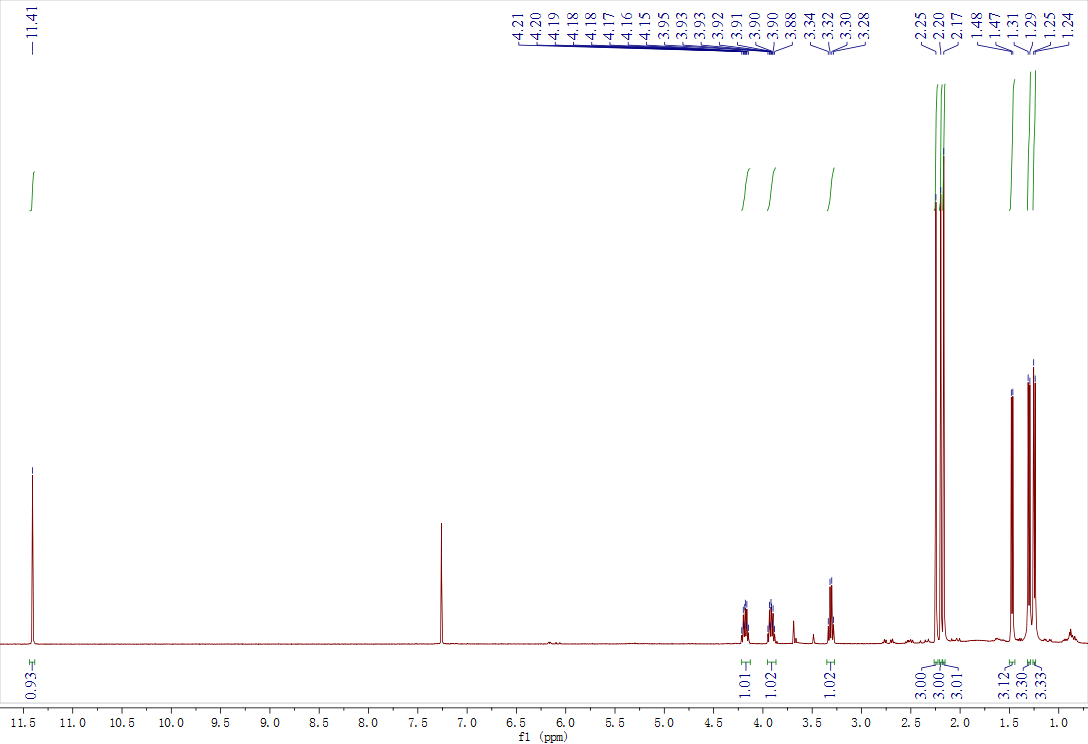


# Figure S1. ^1^H NMR spectrum (400 MHz) of compound 1 in CDCl_3_

**
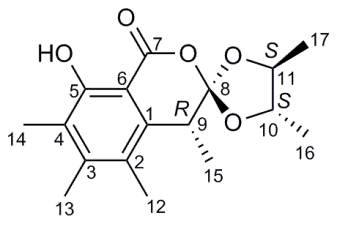

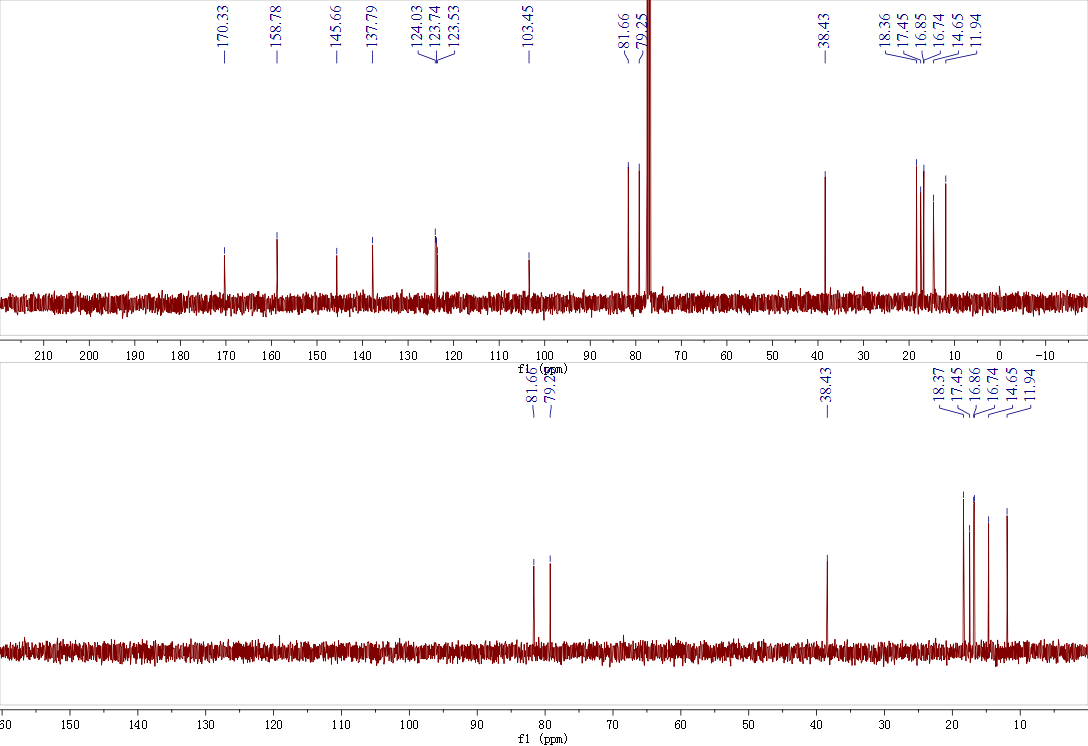
**

# Figure S2. ^13^C NMR spectrum (100 MHz) of compound 1 in CDCl_3_

**
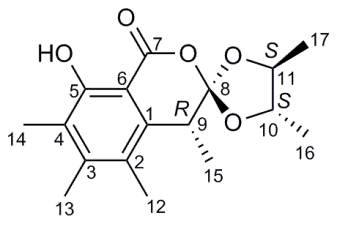

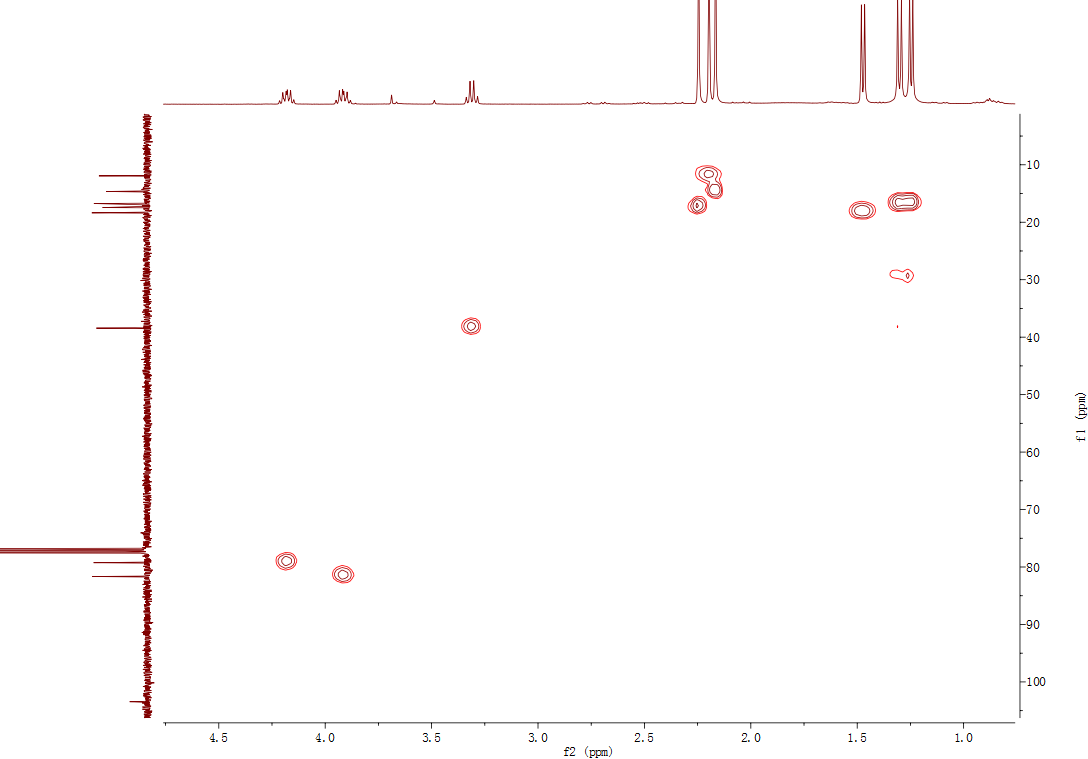
**

# Figure S3. HSQC spectrum of compound 1 in CDCl_3_


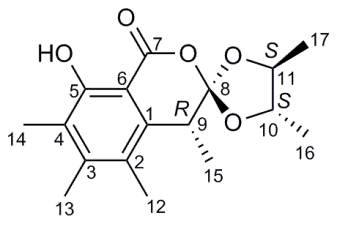

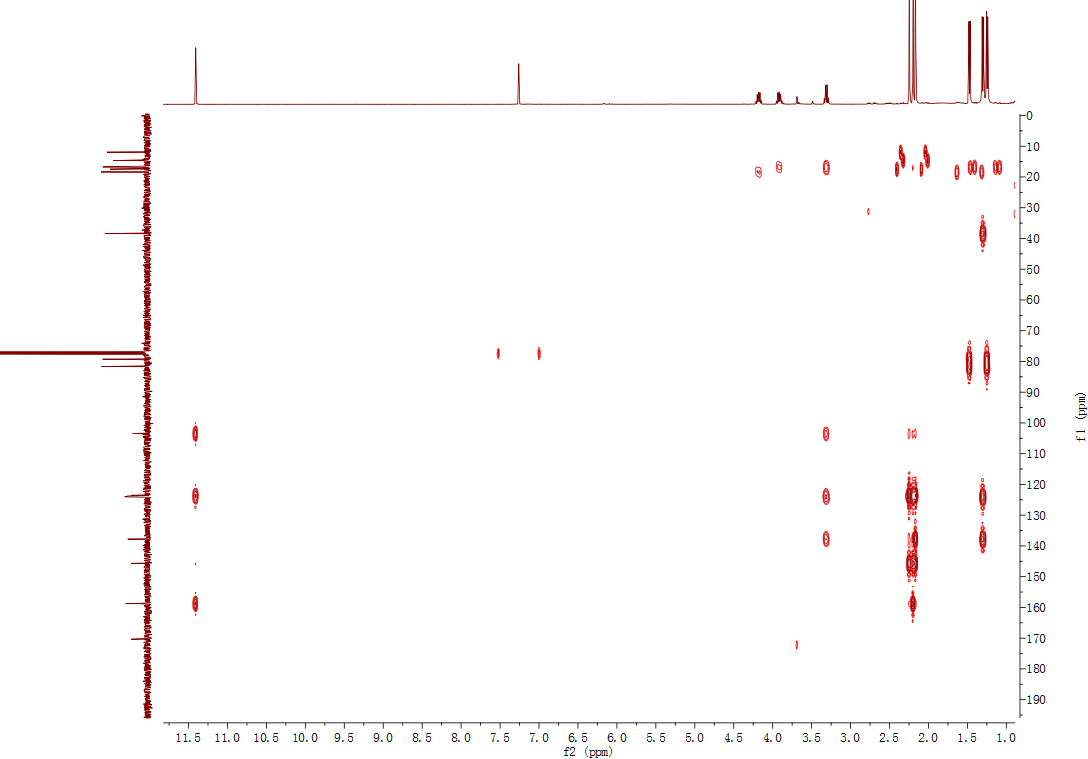
**Figure S4**. HMBC spectrum of compound **1** in CDCl_3_

**
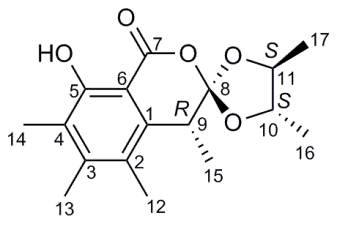

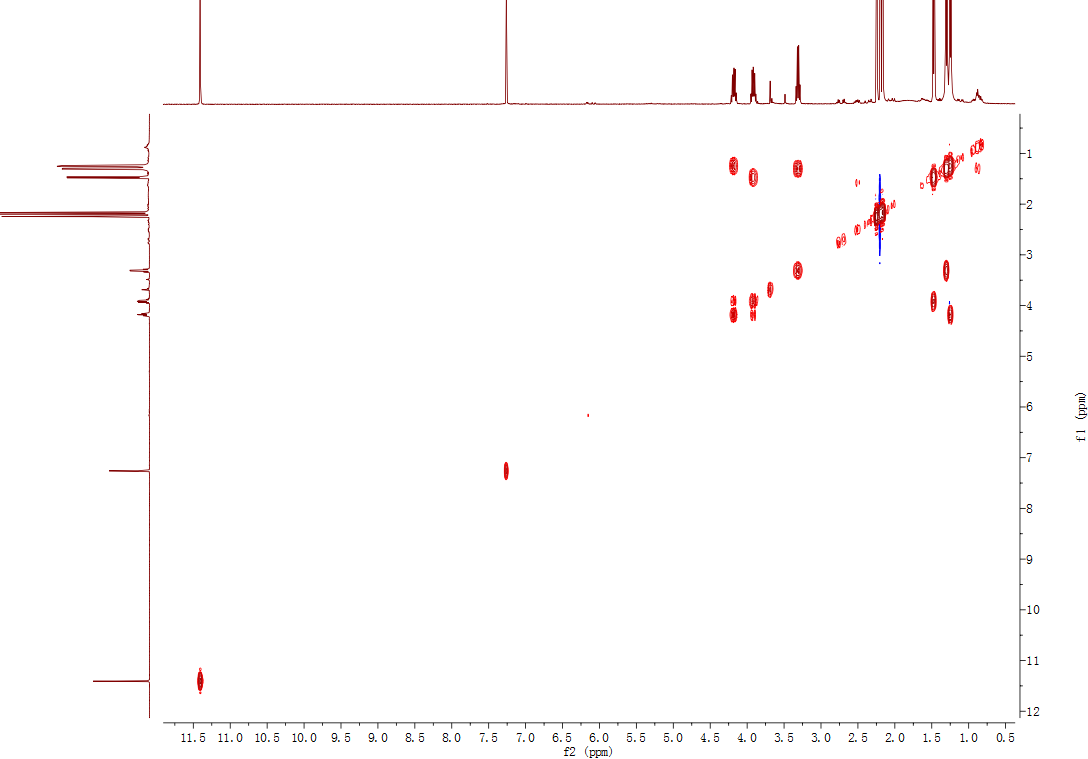
**

# Figure S5. ^1^H-^1^H COSY spectrum of compound 1 in CDCl_3_


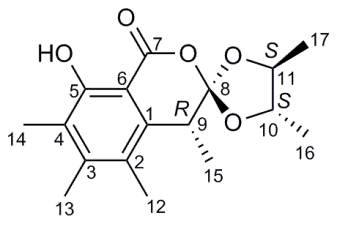
**
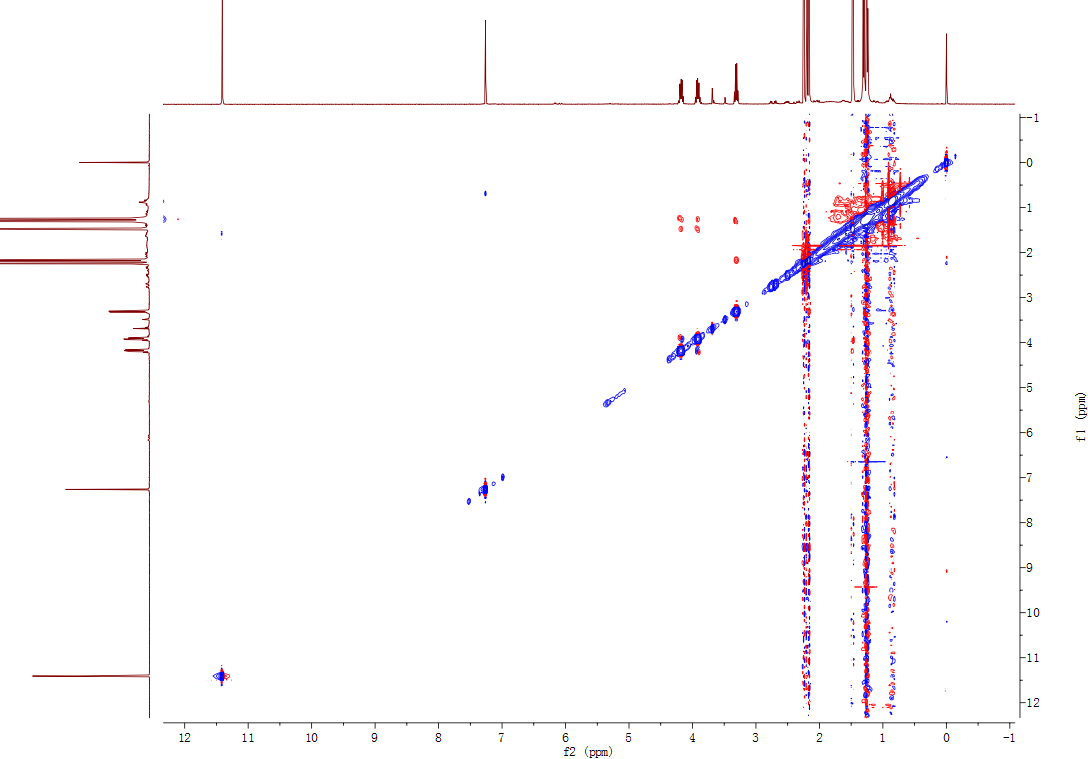
****Figure S6**. NOESY spectrum of compound **1** in CDCl_3_

**
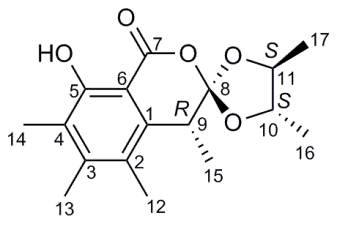
**

# Figure S7. HRESIMS spectrum of compound 1


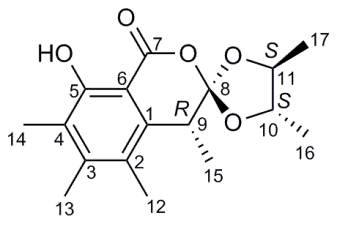


# Figure S8. UV spectrum of compound
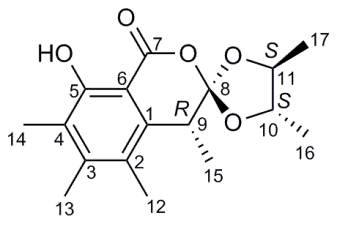
 1

**
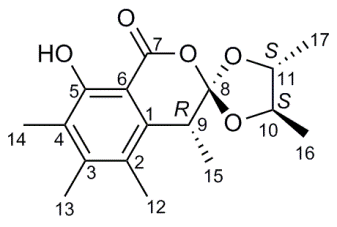
**

# Figure S9. IR spectrum of compound 1
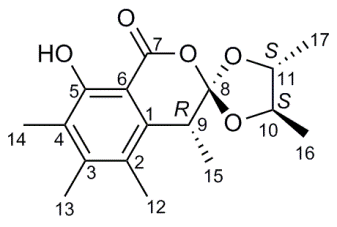

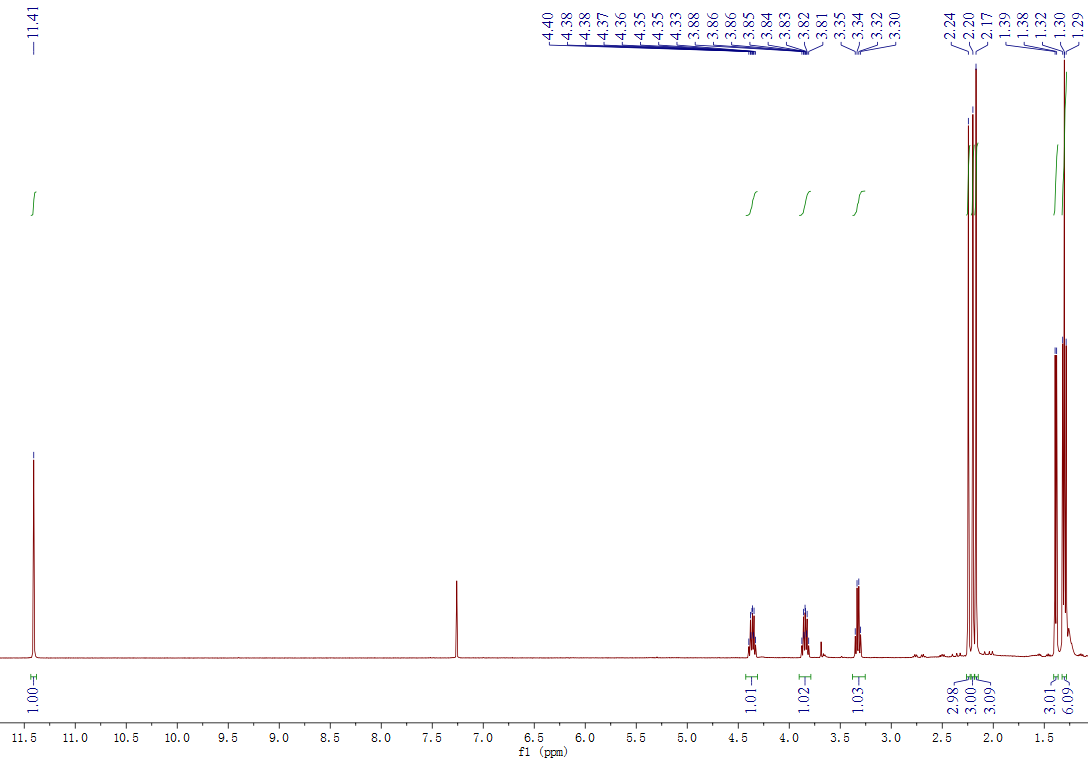
Figure S10. ^1^H NMR spectrum (400 MHz) of compound 2 in CDCl_3_


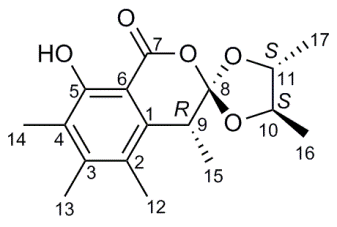
 **
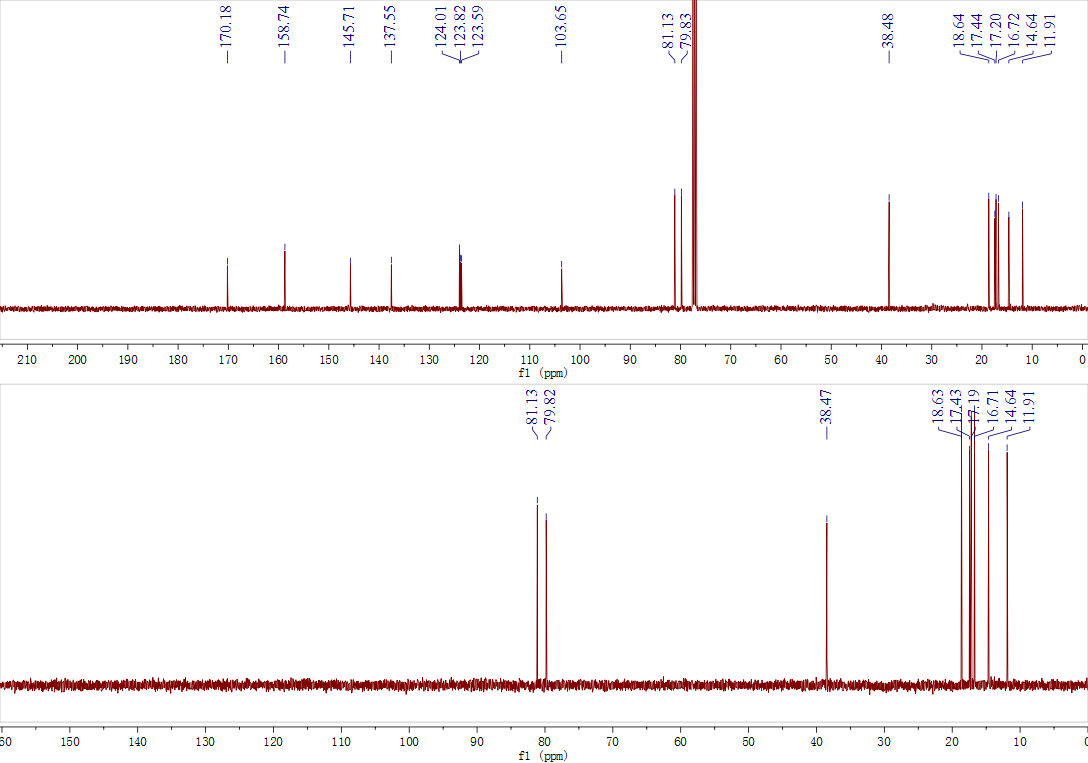
**

# Figure S11. ^13^C NMR spectrum (100 MHz) of compound 2 in CDCl_3_

#
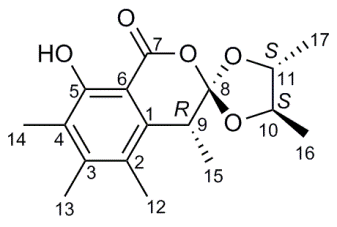

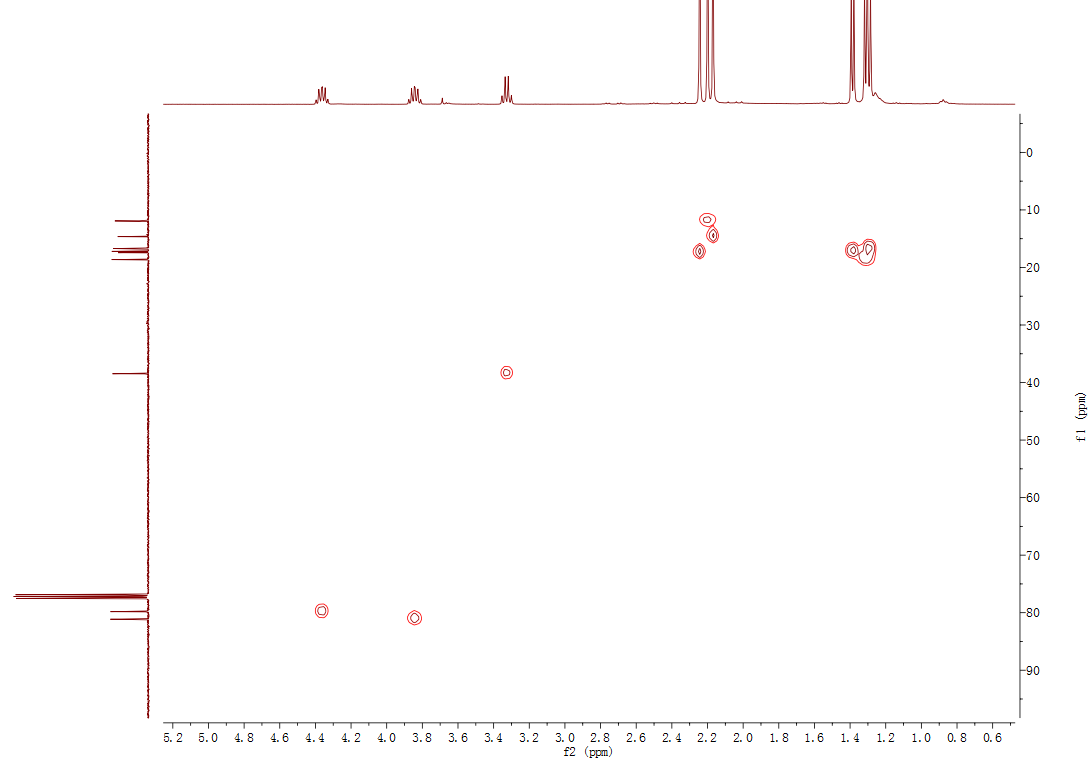
Figure S12. HMQC spectrum of compound 2 in CDCl_3_

#
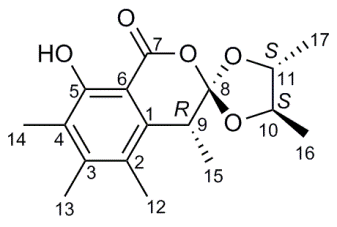

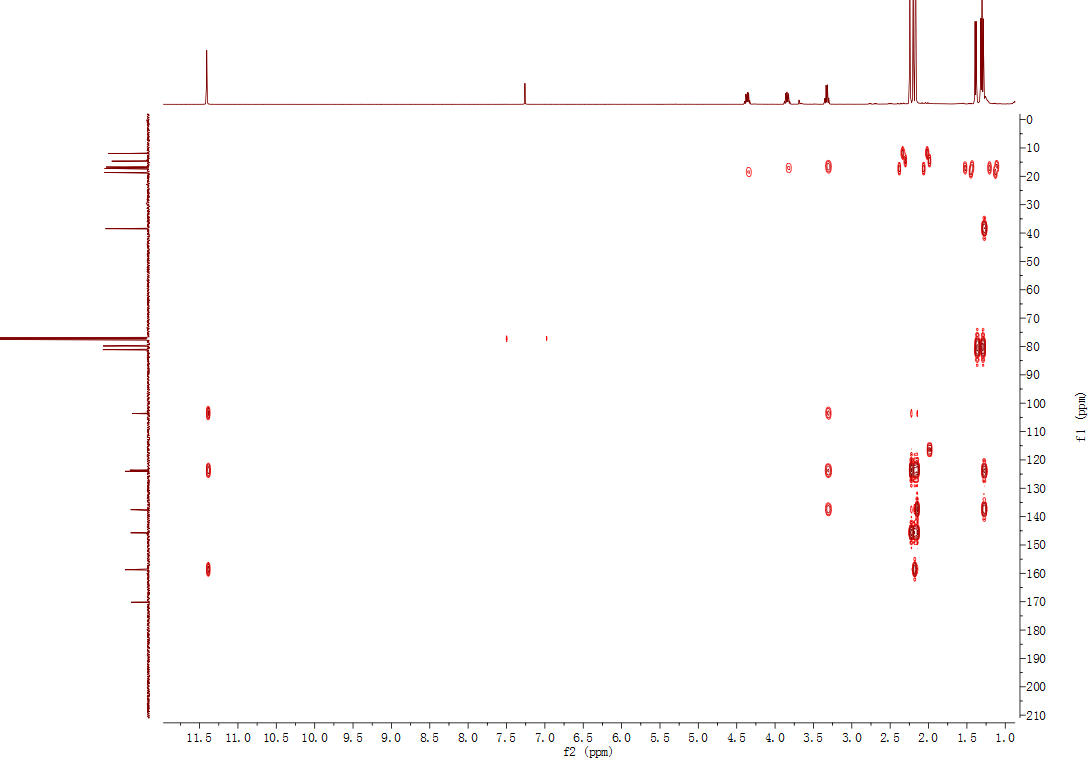
Figure S13. HMBC spectrum of compound 2 in CDCl_3_


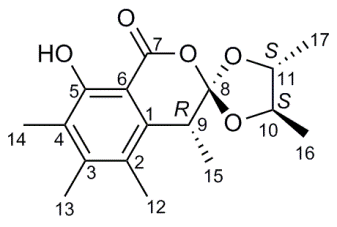

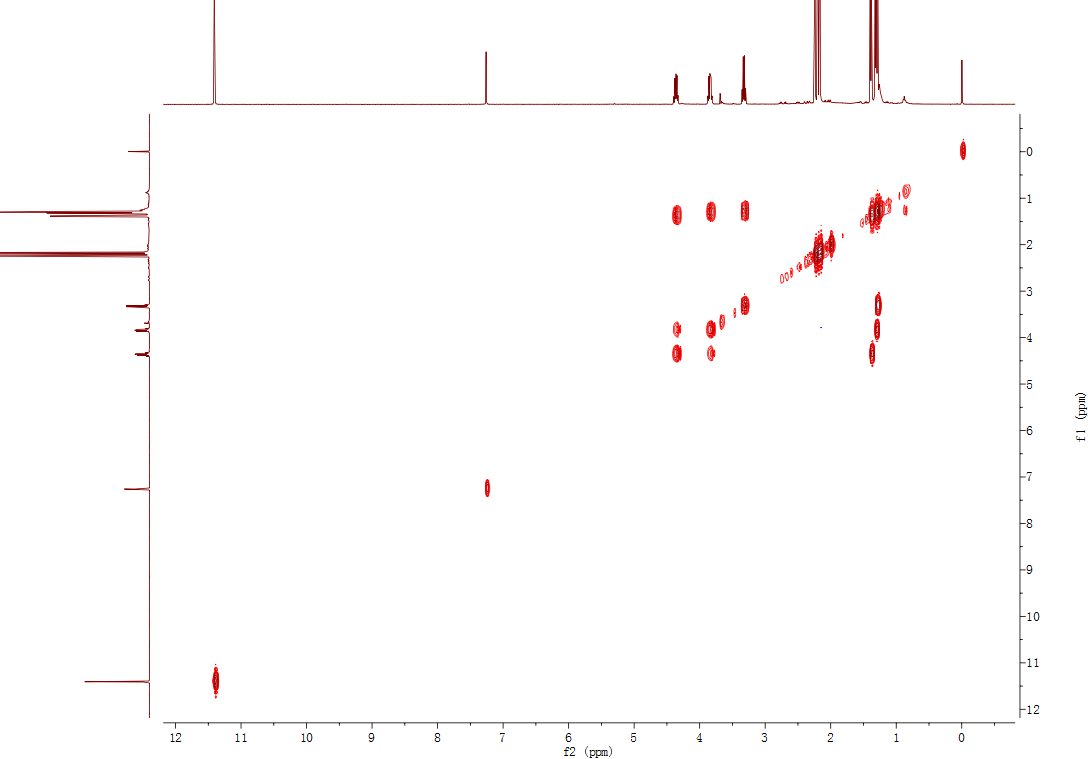


# Figure S14. ^1^H-^1^H COSY spectrum of compound 2 in CDCl_3_

**
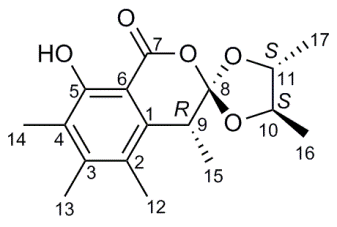

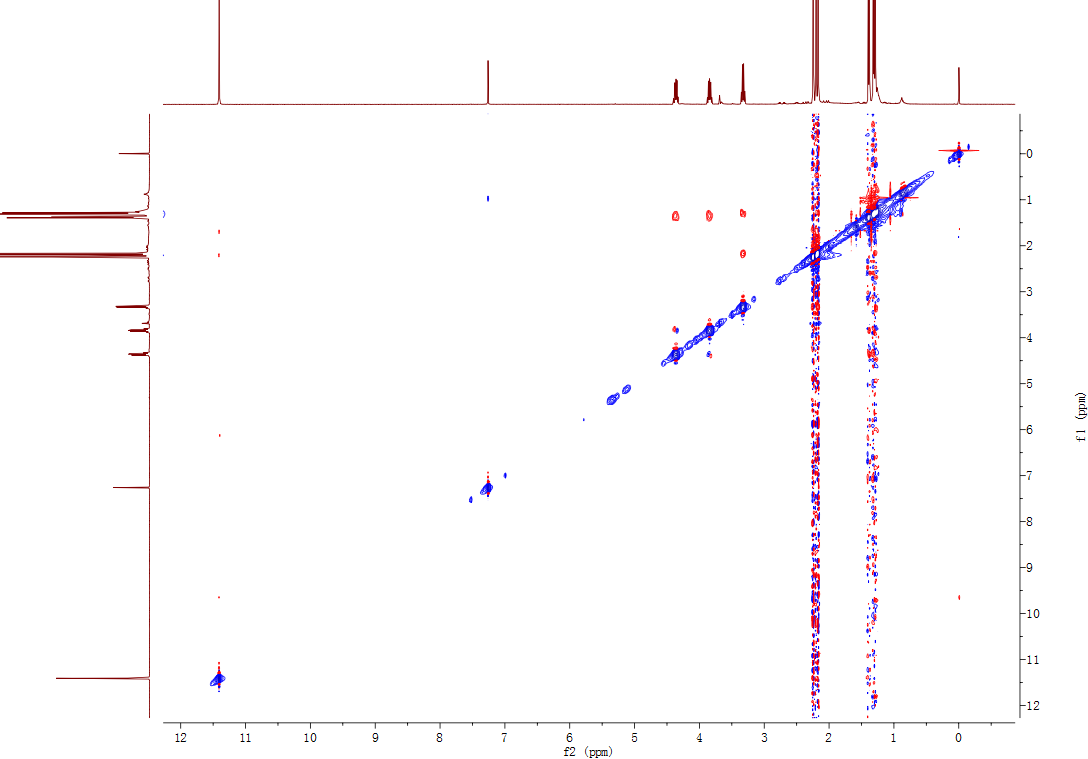
**

# Figure S15. NOESY spectrum of compound 2 in CDCl_3_

**
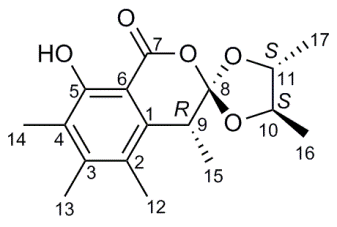
**

# Figure S16. HRESIMS spectrum of compound 2


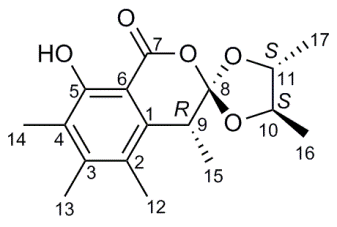


# Figure S17. UV spectrum of compound 2

**
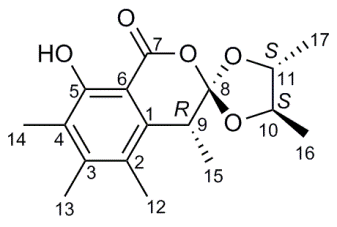
**

# Figure S18. IR spectrum of compound 2

# Figure S19. Crystal packing of compound 1

# Figure S20. Crystal packing of compound 2
