# Supplementary material for: (±)-Peniorthoesters A and B, Two Pairs of Novel Spiro-Orthoester en-antiomers With an Unusual 1,4,6-Trioxaspi-ro[4.5]decane-7-One Unit From Penicillium minioluteum
Source: Front Chem. 2018 Dec 7;6:605. doi: 10.3389/fchem.2018.00605 (PMC6292945; doi:10.3389/fchem.2018.00605)
Supplement: Supplementary file 2 [file Data_Sheet_1.PDF]

(±)-Peniorthoesters A and B, Two Pairs of Novel Spiro-orthoester enantiomers with an unusual 1,4,6-trioxaspiro[4.5]decane-7-one unit from *Penicillium minioluteum*†

Xiaorui Liu,‡ Chunmei Chen,‡ Yinyu Zheng, Mi Zhang, Junjun Liu, Qun Zhou, Jianping Wang, Zengwei Luo, Hucheng Zhu,* and Yonghui Zhang*

Hubei Key Laboratory of Natural Medicinal Chemistry and Resource Evaluation, School of Pharmacy, Tongji Medical College, Huazhong University of Science and Technology, Wuhan 430030, China

*† Electronic Supplementary Information (ESI) available: Full NMR, HRESIMS, UV, and IR spectra of **1** and **2**; detailed of the ECD calculations of **1** and **2**; X-ray data of **1** and **2** (PDF); and crystallographic data (CIF) are included.*

Corresponding Authors' Emails:

zhangyh@mails.tjmu.edu.cn (Y.Z.); zhuhucheng@hust.edu.cn (H.Z.)

‡ These authors contributed equally to this work.

Contents

Figure S1. ^{1}H NMR spectrum (400 MHz) of compound **1** in $CDCl_3$ 3

Figure S2. ^{13}C NMR spectrum (100 MHz) of compound **1** in $CDCl_3$ 3

Figure S3. HSQC spectrum of compound **1** in $CDCl_3$ 4

Figure S4. HMBC spectrum of compound **1** in $CDCl_3$ 4

Figure S5. ^{1}H-^{1}H COSY spectrum of compound **1** in $CDCl_3$ 5

Figure S6. NOESY spectrum of compound **1** in $CDCl_3$ 5

Figure S7. HRESIMS spectrum of compound **1** 6

Figure S8. UV spectrum of compound **1** 6

Figure S9. IR spectrum of compound **1** 7

Figure S10. ^{1}H NMR spectrum (400 MHz) of compound **2** in $CDCl_3$ 7

Figure S11. ^{13}C NMR spectrum (100 MHz) of compound **2** in $CDCl_3$ 8

Figure S12. HMQC spectrum of compound **2** in $CDCl_3$ 8

Figure S13. HMBC spectrum of compound **2** in $CDCl_3$ 9

Figure S14. ^{1}H-^{1}H COSY spectrum of compound **2** in $CDCl_3$ 9

Figure S15. NOESY spectrum of compound **2** in $CDCl_3$ 10

Figure S16. HRESIMS spectrum of compound **2** 10

Figure S17. UV spectrum of compound **2** 11

Figure S18. IR spectrum of compound **2** 11

Figure S19. Crystal packing of compound **1** 12

Figure S20. Crystal packing of compound **2** 12

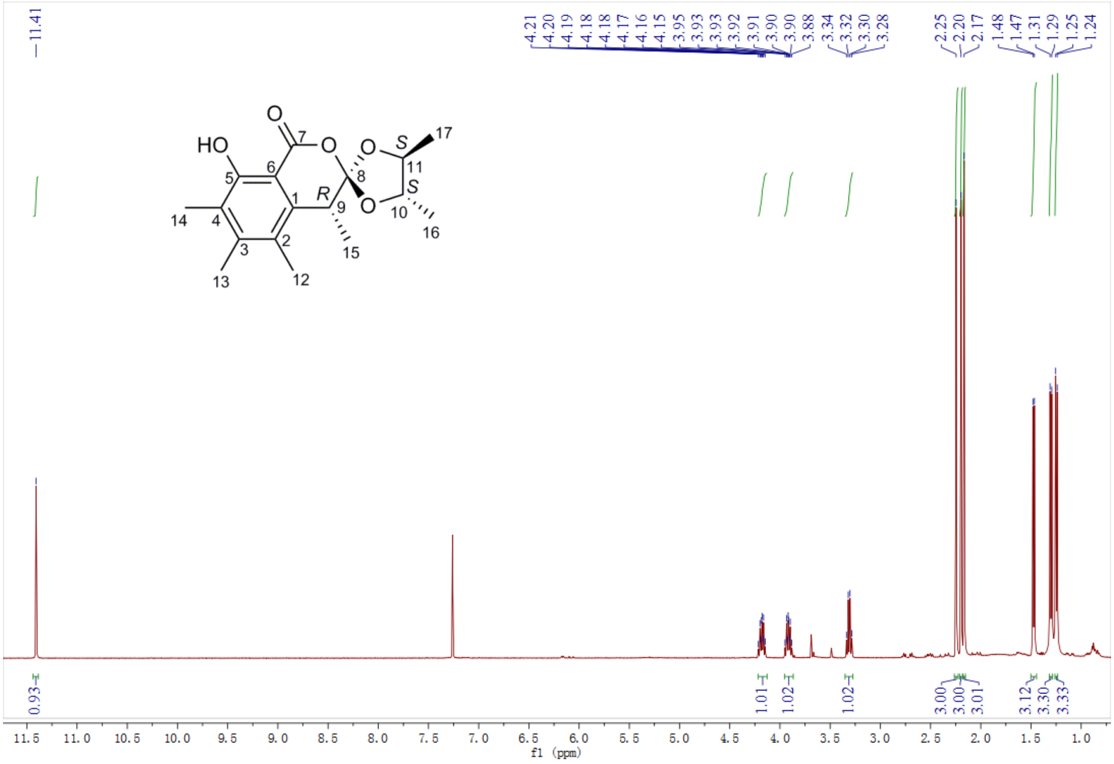

Figure S1. ^{1}H NMR spectrum (400 MHz) of compound **1** in $CDCl_3$

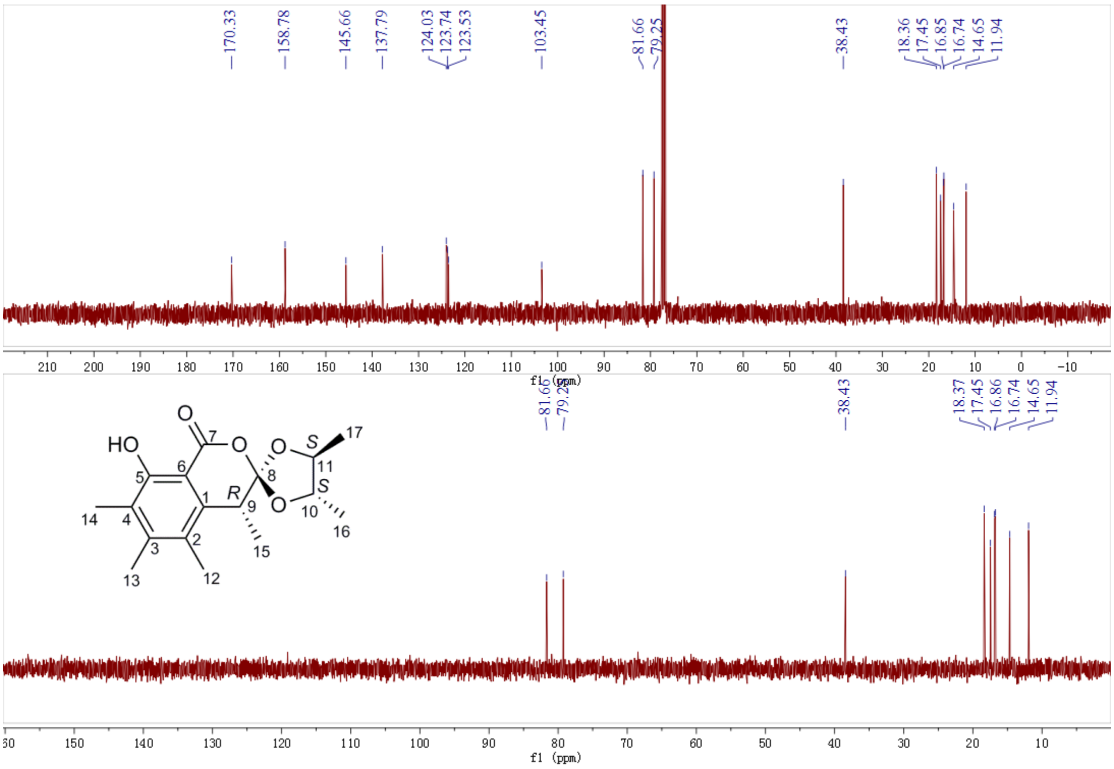

Figure S2. ^{13}C NMR spectrum (100 MHz) of compound **1** in $CDCl_3$

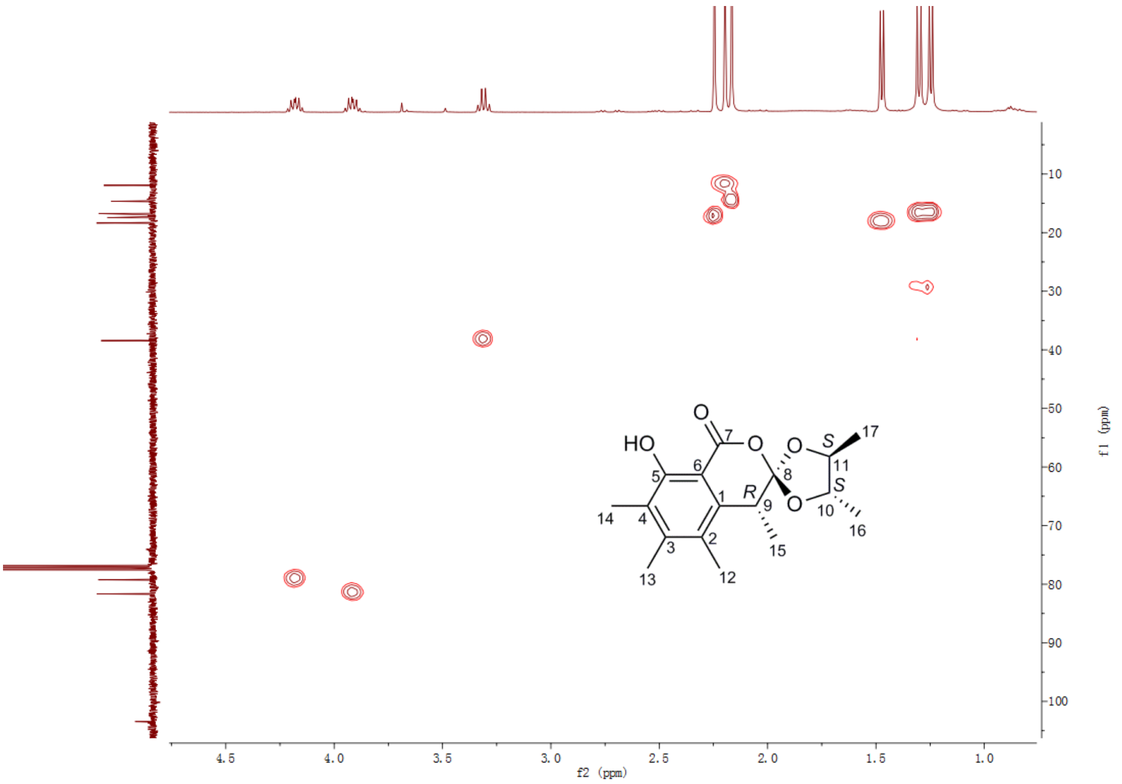

Figure S3. HSQC spectrum of compound **1** in $CDCl_3$

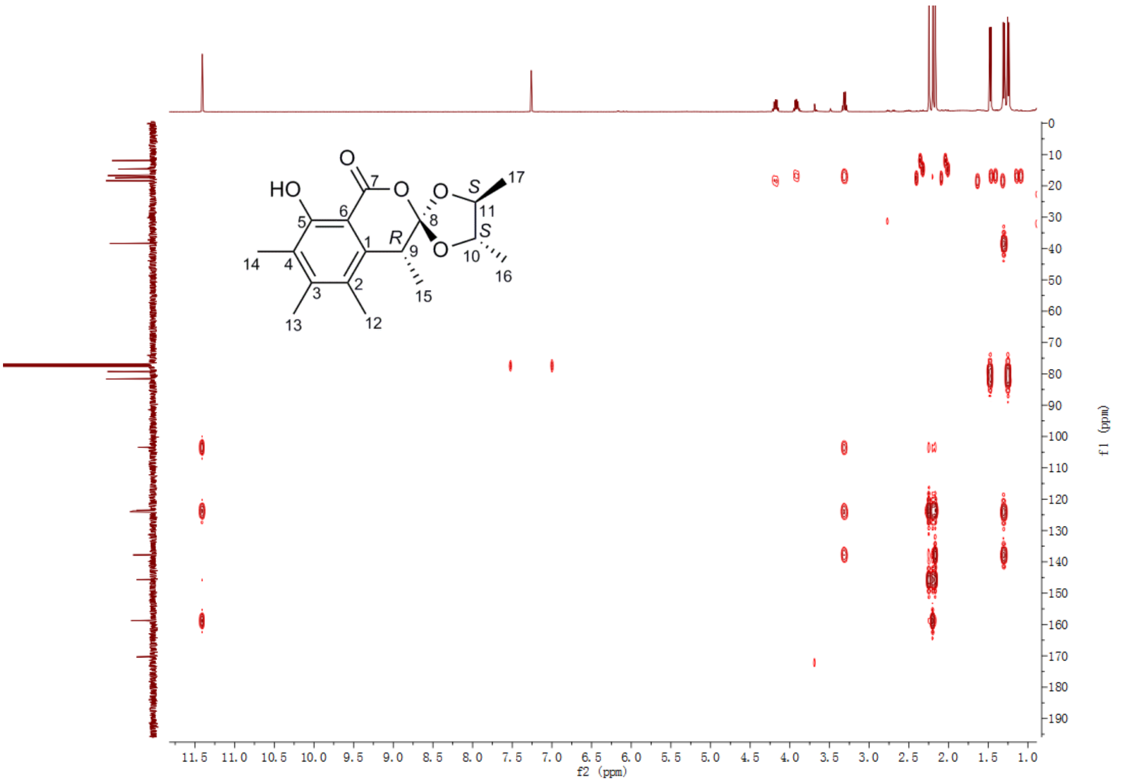

Figure S4. HMBC spectrum of compound **1** in $CDCl_3$

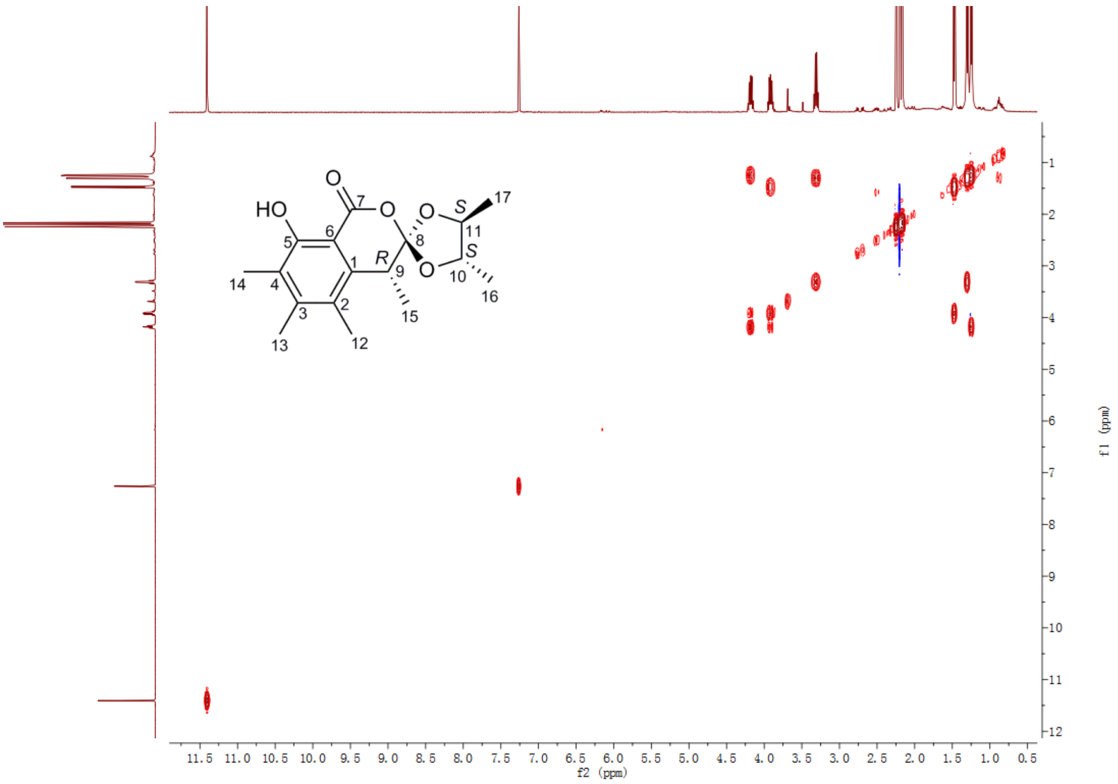

Figure S5. ^{1}H-^{1}H COSY spectrum of compound **1** in $CDCl_3$

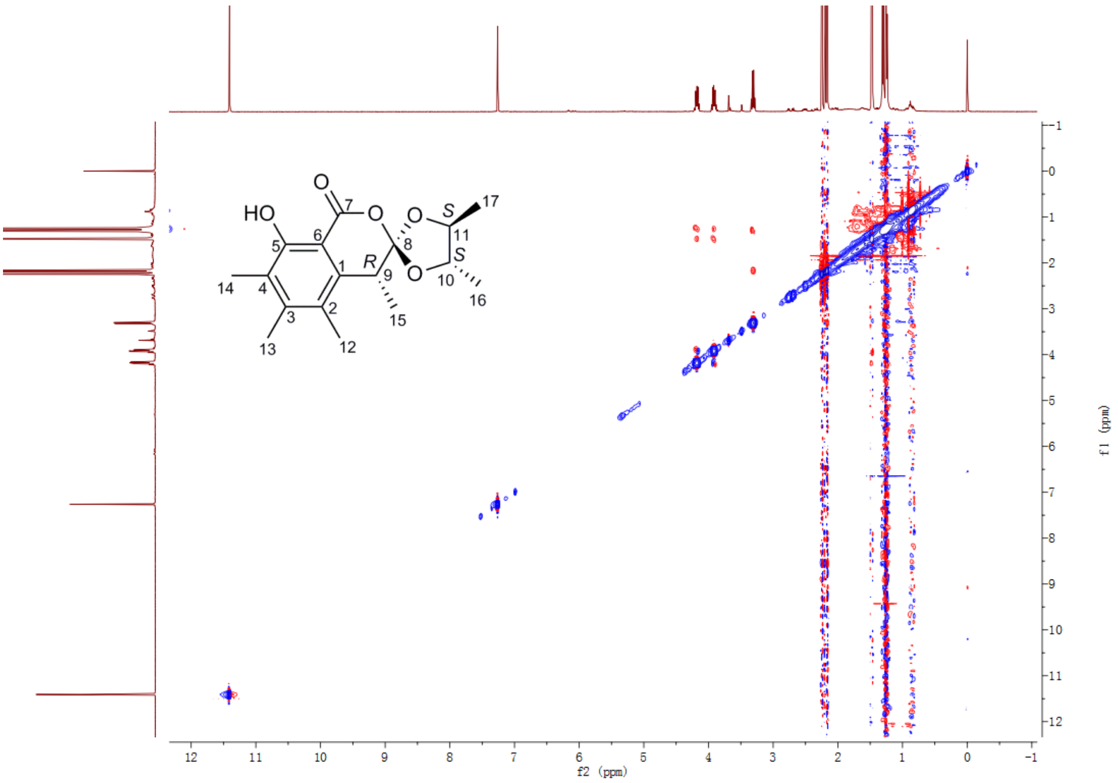

Figure S6. NOESY spectrum of compound **1** in $CDCl_3$

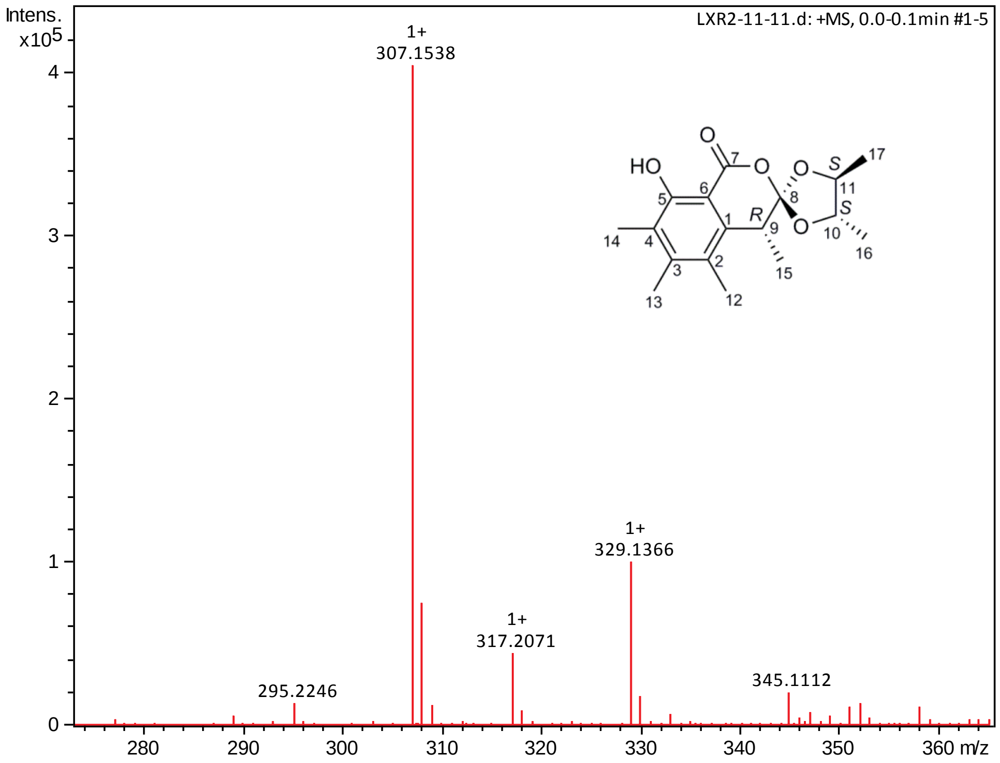

Figure S7. HRESIMS spectrum of compound **1**

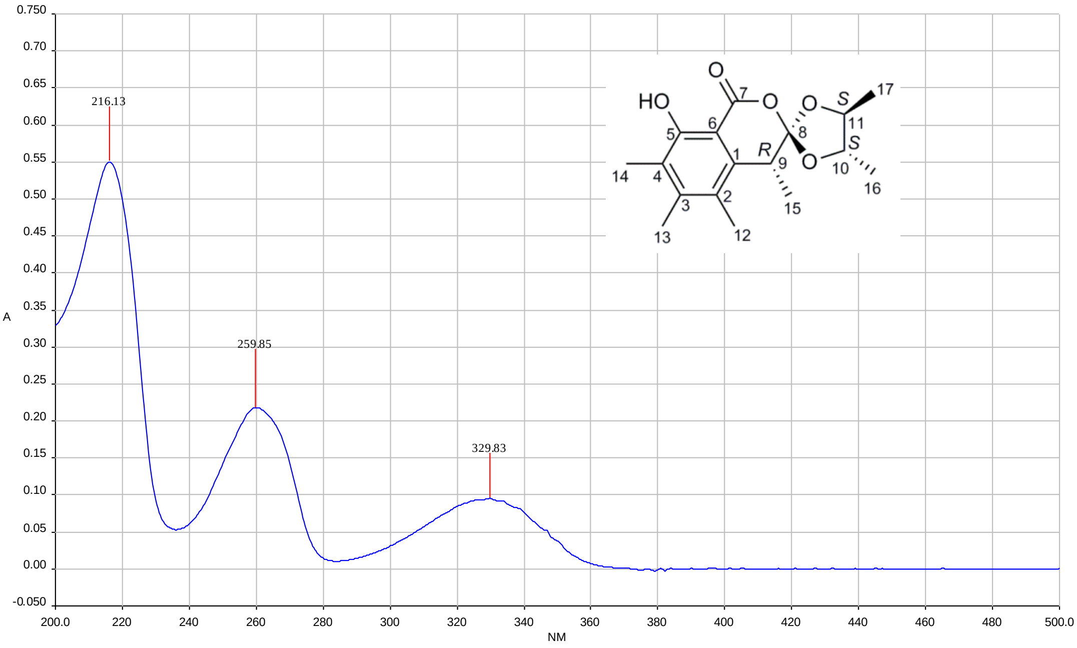

Figure S8. UV spectrum of compound **1**

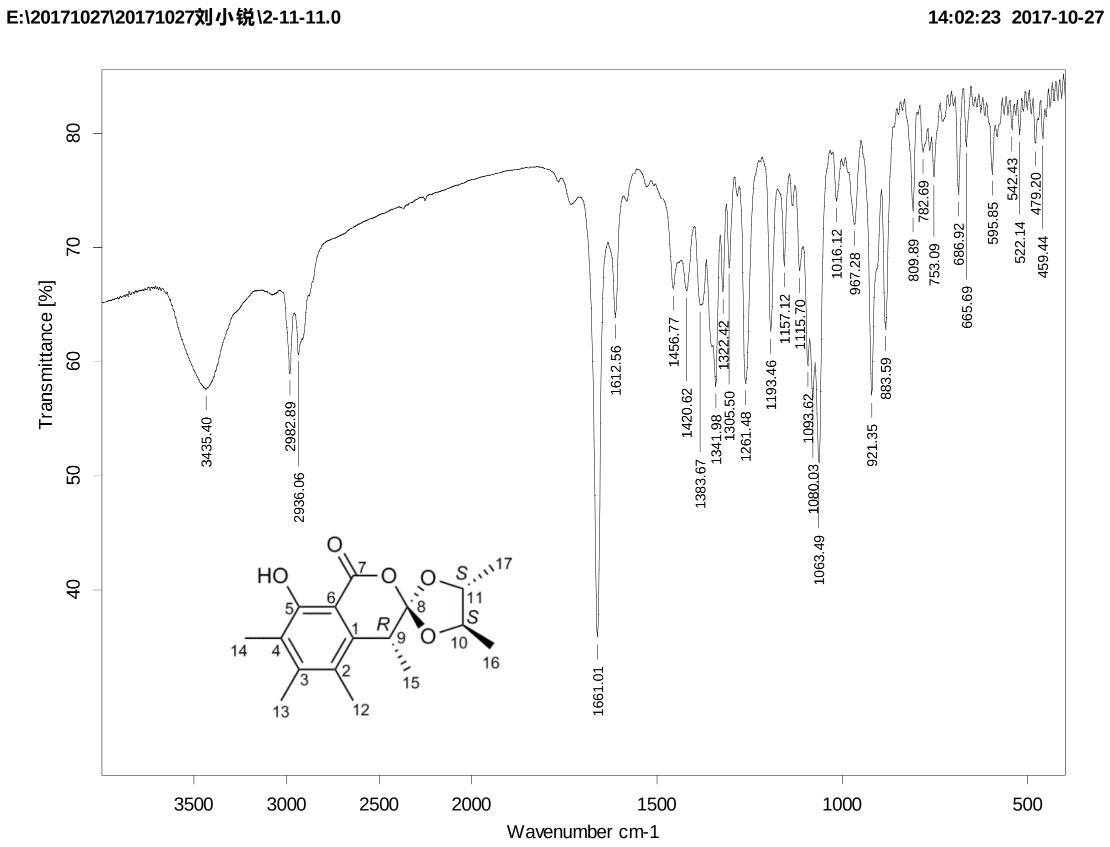

Figure S9. IR spectrum of compound
1

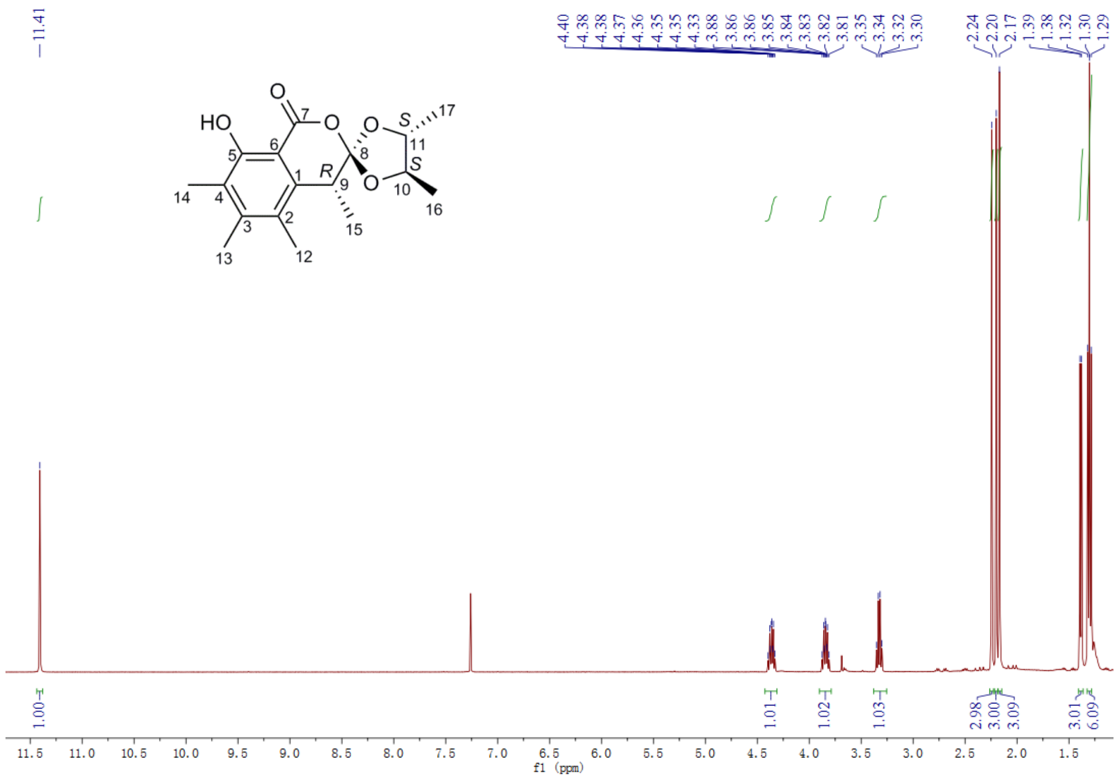

Figure S10. ^{1}H NMR spectrum (400 MHz) of compound **2** in $CDCl_3$

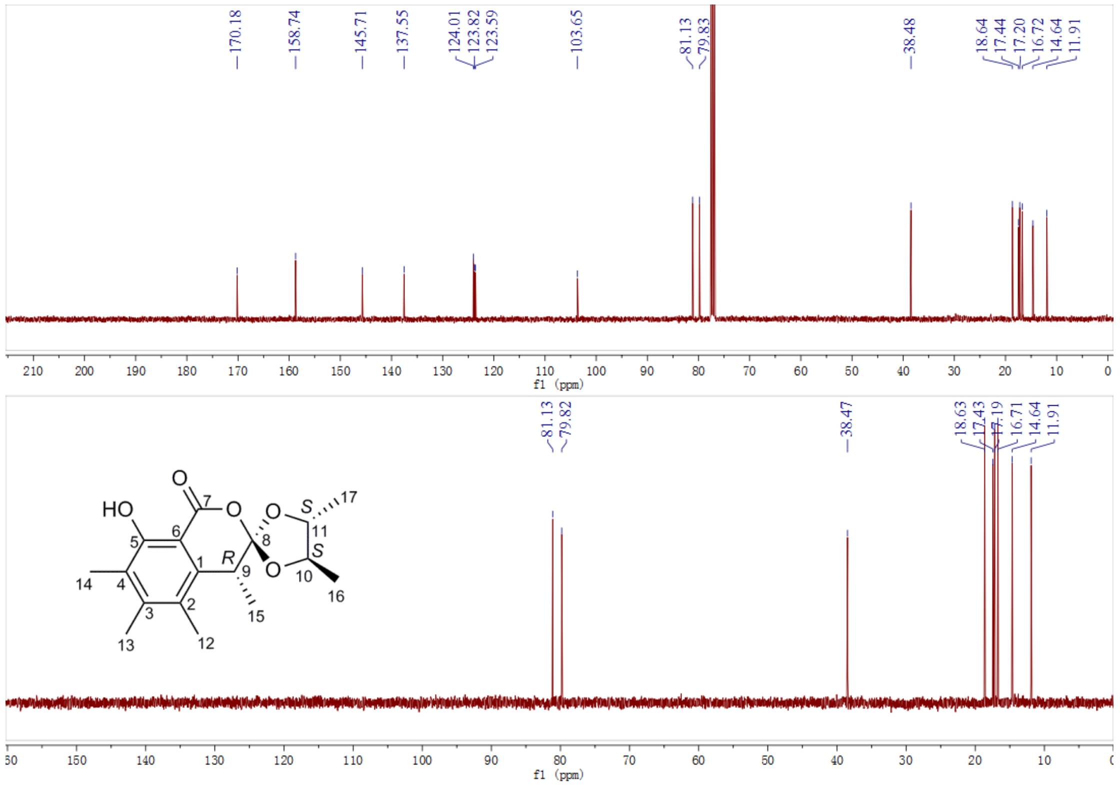

Figure S11. ^{13}C NMR spectrum (100 MHz) of compound **2** in $CDCl_3$

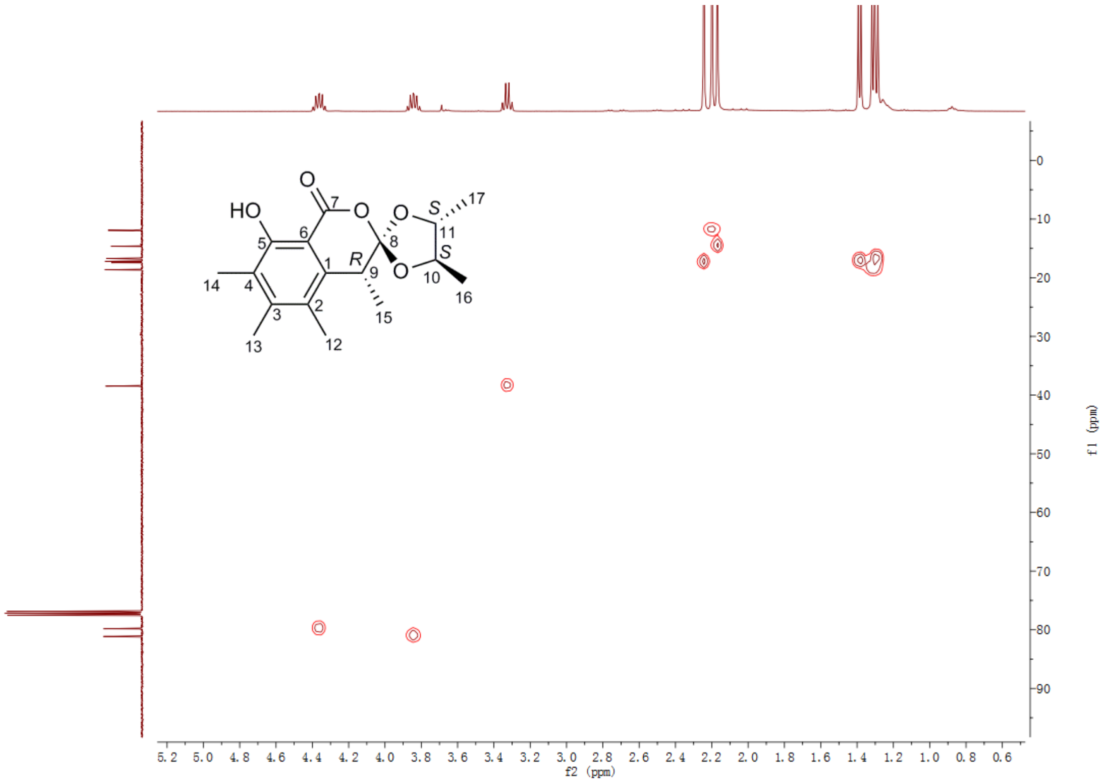

Figure S12. HMQC spectrum of compound **2** in $CDCl_3$

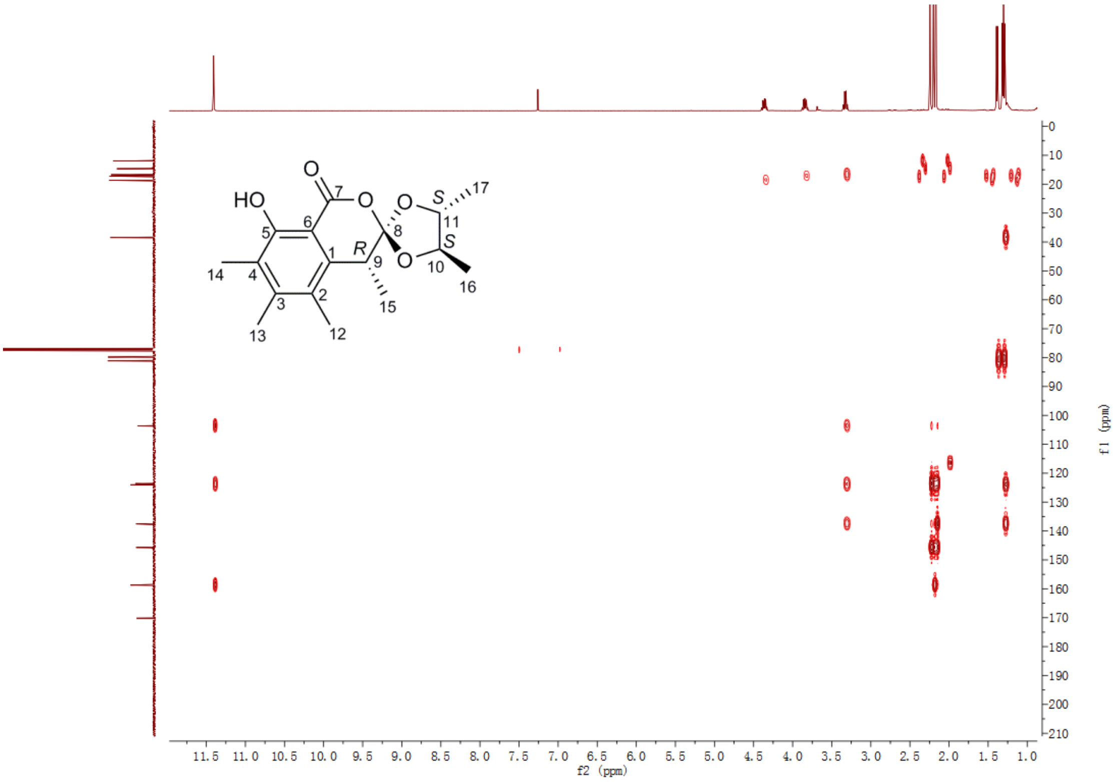

Figure S13. HMBC spectrum of compound **2** in $CDCl_3$

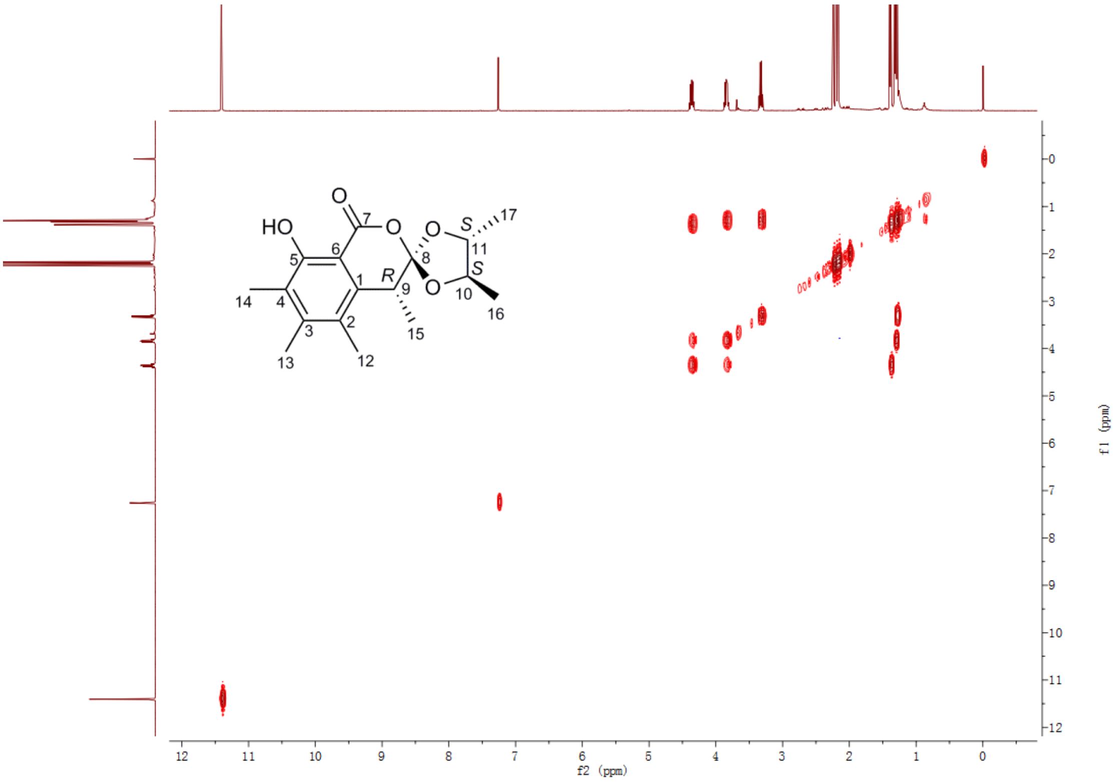

Figure S14. ^1H-^1H COSY spectrum of compound **2** in $CDCl_3$

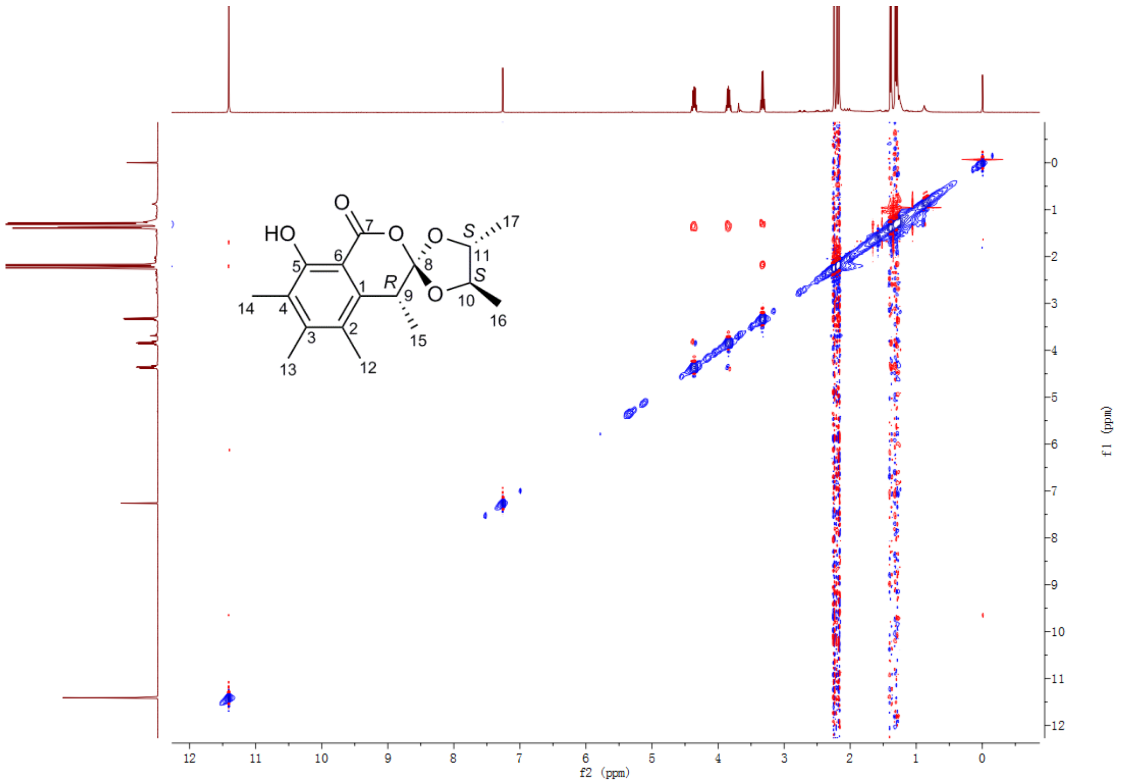

Figure S15. NOESY spectrum of compound **2** in $CDCl_3$

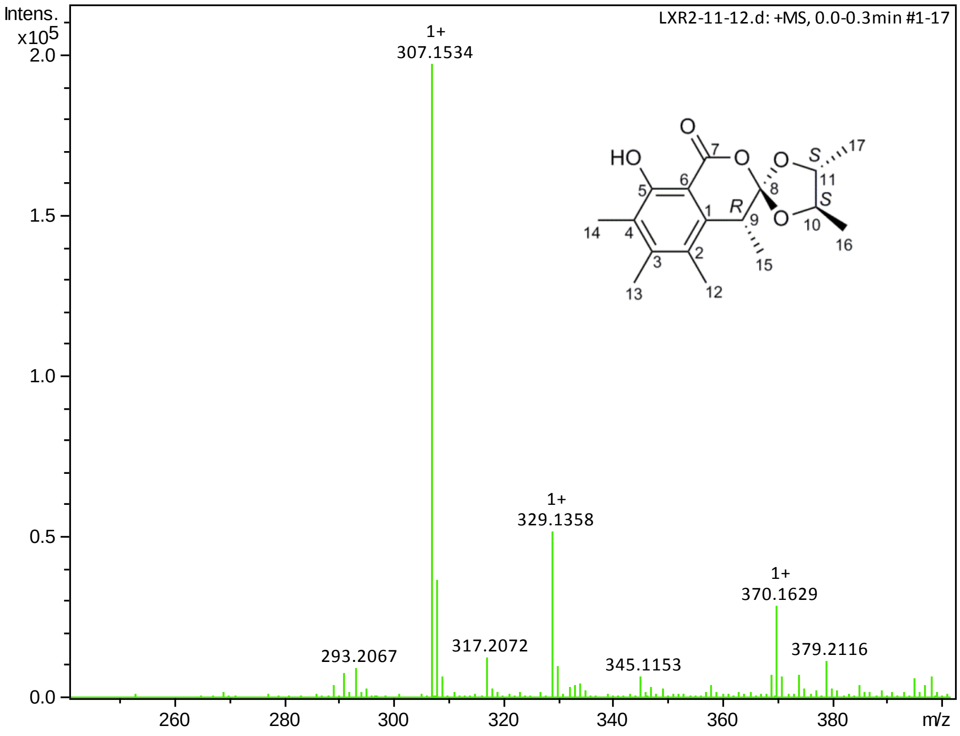

Figure S16. HRESIMS spectrum of compound **2**

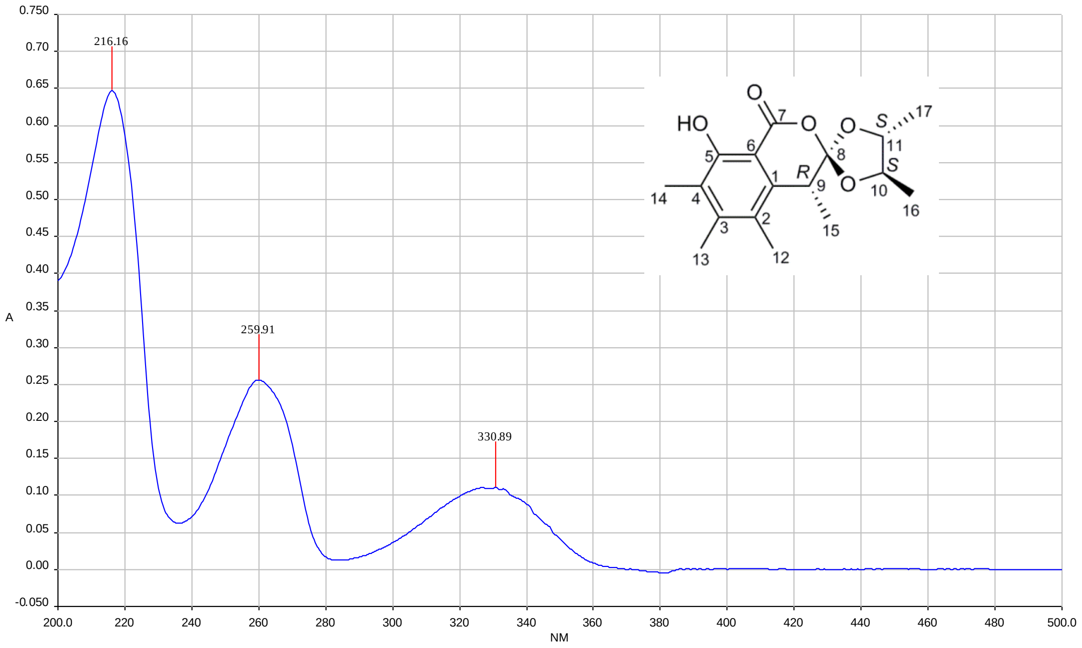

Figure S17. UV spectrum of compound **2**

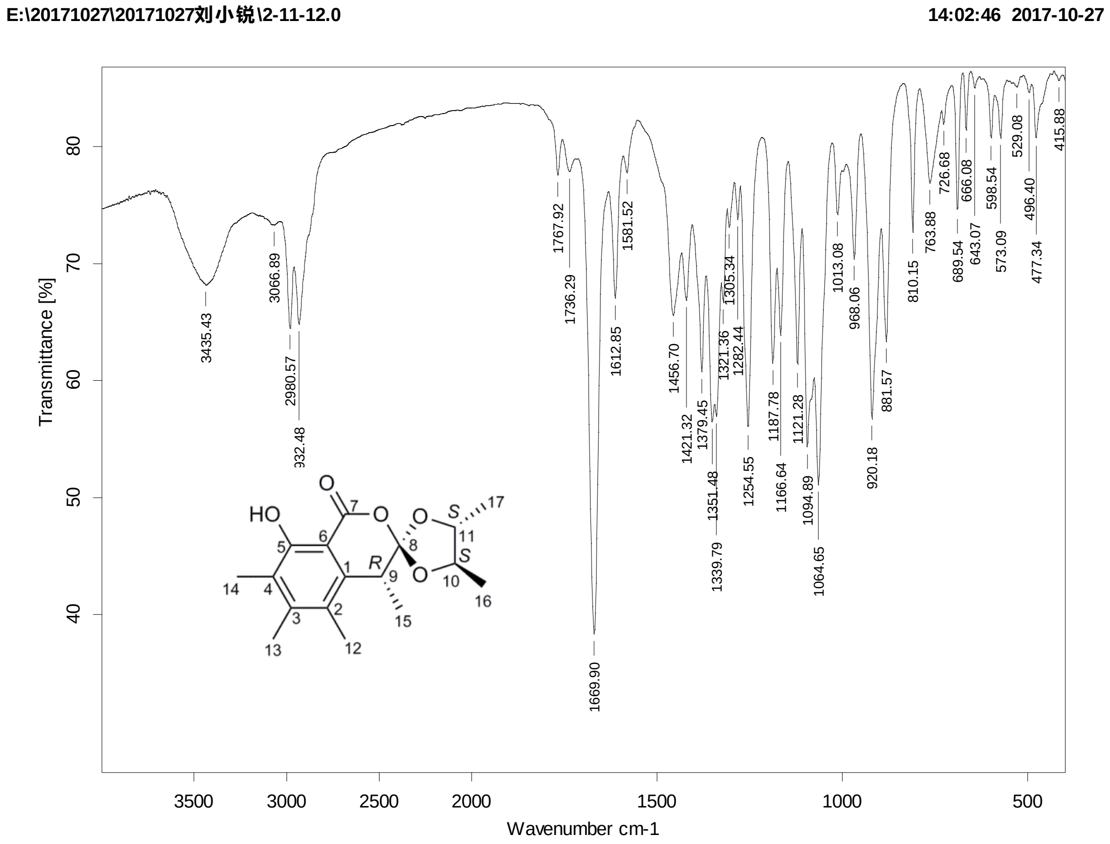

Figure S18. IR spectrum of compound **2**

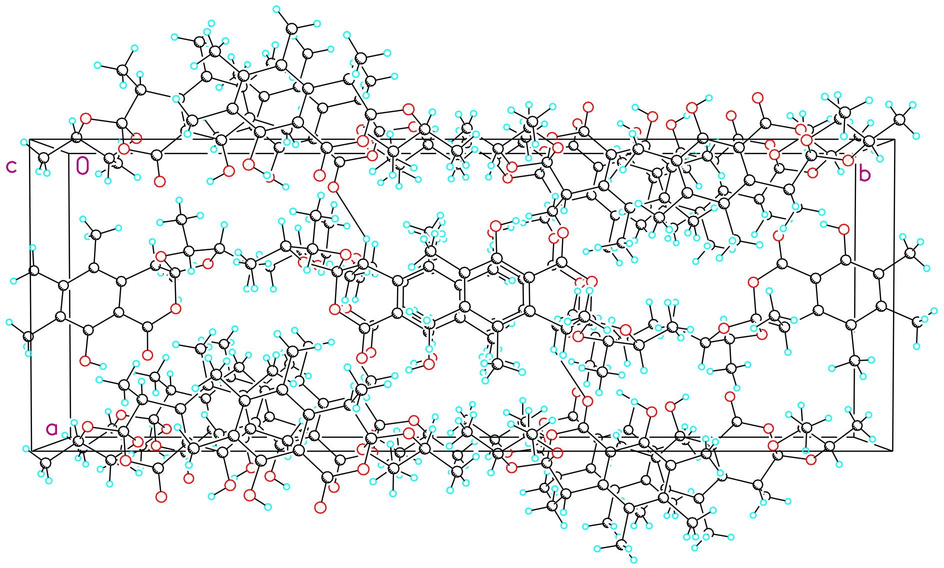

Figure S19. Crystal packing of compound **1**

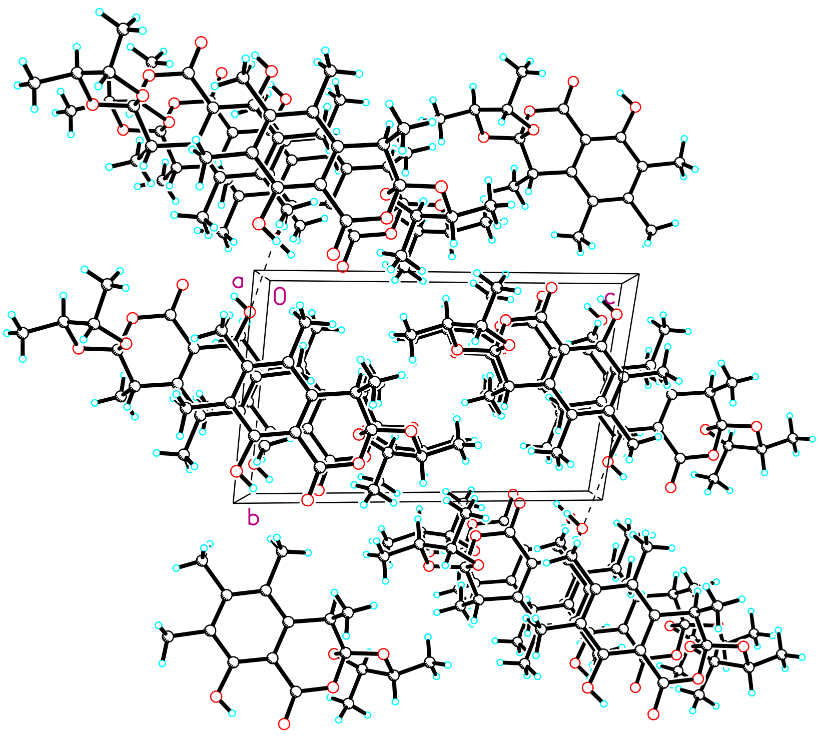

Figure S20. Crystal packing of compound **2**
